# Cost-effectiveness of Universal School- and Community-Based Vision Testing Strategies to Detect Amblyopia in Children in Ontario, Canada

**DOI:** 10.1001/jamanetworkopen.2022.49384

**Published:** 2023-01-04

**Authors:** Afua Oteng Asare, Daphne Maurer, Agnes M. F. Wong, Natasha Saunders, Wendy J. Ungar

**Affiliations:** 1 Institute of Health Policy, Management and Evaluation, University of Toronto, Toronto, Ontario, Canada; 2 Program of Neurosciences and Mental Health, The Hospital for Sick Children Research Institute, Toronto, Ontario, Canada; 3 ICES, Toronto, Ontario, Canada; 4 John Moran Eye Center, Department of Ophthalmology and Visual Sciences, University of Utah, Salt Lake City; 5 Department of Psychology, Neuroscience and Behaviour, McMaster University, Hamilton, Ontario, Canada; 6 Department of Ophthalmology and Vision Sciences, University of Toronto, Toronto, Ontario, Canada; 7 Program of Child Health Evaluative Sciences, The Hospital for Sick Children Research Institute, Toronto, Ontario, Canada; 8 Department of Pediatrics, University of Toronto, Toronto, Ontario, Canada

## Abstract

**Importance:**

Screening for amblyopia in primary care visits is recommended for young children, yet screening rates are poor. Although the prevalence of amblyopia is low (3%-5%) among young children, universal screening in schools and mandatory optometric examinations may improve vision care, but the cost-effectiveness of these vision testing strategies compared with the standard in primary care is unknown.

**Objective:**

To evaluate the relative cost-effectiveness of universal school screening and mandated optometric examinations compared with standard care vision screening in primary care visits in Toronto, Canada, with the aim of detecting and facilitating treatment of amblyopia and amblyopia risk factors from the Ontario government’s perspective.

**Design, Setting, and Participants:**

An economic evaluation was conducted from July 2019 to May 2021 using a Markov model to compare 15-year costs and quality-adjusted life-years (QALYs) between school screening and optometric examination compared with primary care screening in Toronto, Canada. Parameters were derived from published literature, the Ontario Schedule of Benefits and Fees, and the Kindergarten Vision Testing Program. A hypothetical cohort of 25 000 children aged 3 to 5 years was simulated. It was assumed that children in the cohort had irreversible vision impairment if not diagnosed by an optometrist. In addition, incremental costs and outcomes of 0 were adjusted to favor the reference strategy. Vision testing programs were designed to detect amblyopia and amblyopia risk factors.

**Main Outcomes and Measures:**

For each strategy, the mean costs per child included the costs of screening, optometric examinations, and treatment. The mean health benefits (QALYs) gained were informed by the presence of vision impairment and the benefits of treatment. Incremental cost-effectiveness ratios were calculated for each alternative strategy relative to the standard primary care screening strategy as the additional cost required to achieve an additional QALY at a willingness-to-pay threshold of $50 000 Canadian dollars (CAD) ($37 690) per QALY gained.

**Results:**

School screening relative to primary care screening yielded cost savings of CAD $84.09 (95% CI, CAD $82.22-$85.95) (US $63.38 [95% CI, US $61.97-$64.78]) per child and an incremental gain of 0.0004 (95% CI, −0.0047 to 0.0055) QALYs per child. Optometric examinations relative to primary care screening yielded cost savings of CAD $74.47 (95% CI, CAD $72.90-$76.03) (US $56.13 [95% CI, $54.95-$57.30]) per child and an incremental gain of 0.0508 (95% CI, 0.0455-0.0561) QALYs per child. At a willingness-to-pay threshold of CAD $50 000 (US $37 690) per QALY gained, school screening and optometric examinations were cost-effective relative to primary care screening in only 20% and 29% of iterations, respectively.

**Conclusions and Relevance:**

In this study, because amblyopia prevalence is low among young children and most children in the hypothetical cohort had healthy vision, universal school screening and optometric examinations were not cost-effective relative to primary care screening for detecting amblyopia in young children in Toronto, Canada. The mean added health benefits of school screening and optometric examinations compared with primary care screening did not warrant the resources used.

## Key Points

**Question:**

Is school screening or optometric examinations to detect amblyopia in kindergarten-aged children cost-effective vs vision screening in primary care visits?

**Findings:**

In this economic evaluation involving a hypothetical cohort of 25 000 children aged 3 to 5 years in Toronto, Canada, school screening relative to primary care screening yielded cost savings of $84.09 Canadian dollars (CAD) ($63.38 US dollars) and an incremental gain of 0.0004 quality-adjusted life-years per child. Optometric examinations relative to primary care screening yielded cost savings of CAD $74.47 (US $56.13) and an incremental gain of 0.0508 quality-adjusted life-years per child.

**Meaning:**

These findings suggest that, because of the low prevalence of amblyopia (5.8%) among young children in the population, school screening and optometric examinations are not cost-effective relative to primary care screening for detecting amblyopia in kindergarten-aged children in Toronto, Canada.

## Teaser

This economic evaluation assesses the relative cost-effectiveness of school screening and optometric examinations compared with vision screening during primary care visits among a hypothetical cohort of children aged 3 to 5 years in Toronto, Canada.

## Introduction

The prevalence of amblyopia (lazy eye) ranges between 3% and 5% in young children depending on the population tested and the definition adopted.^1,2,3,4,5^ Approximately 10% of children aged 3 to 6 years have conditions that put them at high risk of developing amblyopia (ie, strabismus, anisometropia, and opacities of the ocular media caused by cataracts and drooping eyelids).^6,7,8,9,10,11^ If risk factors are identified and treated early, lifelong vision impairment from amblyopia can be prevented.^12,13,14^

To detect amblyopia early, vision screening in well-child visits by primary care physicians and comprehensive eye examinations by eye care professionals are recommended throughout childhood.^14,15,16,17,18,19,20,21,22^ The Canadian Paediatric Society recommends 1 vision screening test by a primary care physician at ages 3 months, 6 months, 9 months, and 12 months and annually from ages 3 years to 6 years.^16^ Similar guidelines have been adopted by the American Academy of Pediatrics.^19^ In addition, the Canadian Association of Optometrists, like the American Optometric Association, recommends 2 comprehensive eye examinations by eye care professionals by age 6 years and annual exams until age 18 years.^15,17,23^

In Ontario, Canada’s largest province with approximately 3 million children, universal funding for children’s annual comprehensive eye examinations and vision screening as part of well-child visits is provided through provincial health insurance.^24,25^ Furthermore, some provincial and federal programs provide coverage for prescription glasses for children who receive social assistance, children who identify as First Nations or Inuit, and all children enrolled in junior kindergarten (aged 4 years).^26,27,28,29^

Despite recommendations and availability of universal funding, the uptake of vision services in Ontario has been poor.^16,30^ Therefore, in 2018, the Ontario Ministry of Health introduced guidelines for administering vision screening in senior kindergartens (children aged 5 years) by public health departments.^31^ Introducing school-based screening has been difficult because of increasing costs and budgeting constraints. As an alternative to underfunded or suboptimally administered universal screening programs, optometric associations in Canada have advocated for mandatory comprehensive eye examinations by optometrists (optometric examinations) throughout childhood.^32^

Considering the budgeting constraints and Ontario’s investments in primary care reform, evidence on the cost-effectiveness of alternative vision testing strategies is important to delivering effective programs, yet data are scarce.^33,34,35,36,37,38^ This study aimed to evaluate the incremental costs and health benefits of public health school screening and optometrist-based vision testing strategies compared with vision screening in well-child visits by primary care physicians with the aim of detecting amblyopia and amblyopia risk factors and facilitating treatment for a child living in Toronto, Canada, from the perspective of the Ontario government.

## Methods

### Study Design

This economic evaluation was conducted from July 2019 to May 2021. The costs and health benefits of 2 alternative universal vision testing strategies (school screening and optometric examinations) to detect and facilitate treatment of amblyopia and amblyopia risk factors in children aged 3 to 5 years in Toronto, Canada, were compared with standard primary care screening from the perspective of the Ontario government. Alternative strategies were compared with standard care rather than each other to provide an analysis that would be most useful to policy and decision makers in the current climate of primary care reforms in Ontario. A hypothetical cohort of 25 000 children was simulated over 15 years in a probabilistic health state transition model. Inputs were obtained from the literature and deidentified patient-level data; therefore, the study was exempted from the need for informed consent by the research ethics board of the Hospital for Sick Children and the University of Toronto. This study followed the Consolidated Health Economic Evaluation Reporting Standards (CHEERS) reporting guideline for economic evaluations.^39^

A number of assumptions were made for this analysis. Important among them was the assumption that children had irreversible vision impairment if not diagnosed by an optometrist. In addition, incremental costs and outcomes of 0 were adjusted to favor the reference strategy.

### Vision Testing Strategies

The reference strategy was universal vision screening in clinics annually from age 3 to 5 years as part of well-child visits by primary care physicians (ie, family physicians and pediatricians), as recommended by the Canadian Paediatric Society.^16^ Primary care physicians could refer children with positive or inconclusive test results to an optometrist for diagnosis and treatment.

The first alternative strategy was a hypothetical universal school-based vision screening (school screening) program modeled after the program planned by the Toronto Public Health Department, consistent with the directive of the Ministry of Health for vision screening in schools.^31,40^ Screening was conducted by contracted screeners trained by a public health nurse.^41^ All children were referred to an optometrist after a positive or inconclusive screening result. The criteria for referral (eAppendix 2 and eTable 1 in Supplement 1) were applied to patient-level data of vision screening test results for Ontario children aged 4 to 5 years to establish the sensitivity and specificity of the screening test in the universal school screening strategy.^40,42^ The second hypothetical alternative strategy was mandated optometric examinations in clinics for all children once between ages 3 years and 5 years per recommendations by the Canadian Association of Optometrists.^17^

### Model

Both the vision testing and the subsequent examination and treatment by an optometrist were modeled (eAppendix 1 and eFigure 1 in Supplement 1). A child entered the model with either healthy vision, amblyopia, or an amblyopia risk factor at age 3 years and could transition to other health states after a year (eAppendix 3 and eFigure 2 in Supplement 1).

A diagnosis of amblyopia or an amblyopia risk factor was based on the accuracy of the clinical assessment by an optometrist, which was assumed to be 95%. Amblyopia and amblyopia risk factors were assumed to cause unilateral (and not bilateral) vision impairment. Children were treated with prescription glasses and additional patching for amblyopia. Children that adhered to a referral incurred the cost of an optometric examination, regardless of whether the test result was true-positive or false-positive. Death occurred based on life expectancy rates obtained from Statistics Canada life tables.^43^ Children exited the model at the end of their 17th year if death did not occur.

### Transition Probabilities

Transition probabilities were derived from published data in the literature^30,33,35,37,44,45,46,47,48,49,50,51,52,53,54,55,56,57,58,59,60,61,62,63,64,65^ and Statistics Canada^43^ and from published and unpublished patient-level data from the Kindergarten Vision Testing Program^42^ (Table 1). The risk of developing amblyopia or experiencing treatment failure increased with increasing age.^44,45,46,47,48,49,50,66^

**Table 1.** Model Parameters, Reference Case Estimates, Parameter Distributions, and Data Sources Used in the Markov Model

Parameter	Age of children, y	Reference case estimate	Parameter distribution	Data source
**Epidemiological data**				
Prevalence of untreated amblyopia	3	0.058	β	Tarczy-Hornoch et al,^54^ 2011
Prevalence of untreated amblyopia risk factor	3	0.202	β	Unpublished KVTP data from Nishimura et al,^42^ 2019
**Probability of events**				
Optometric examinations for children	3-5	0.880	NA	Walkinshaw,^55^ 2011
Vision screening by primary care physicians^a^	3-5	0.610	β	Asare et al,^30^ 2022; Le et al,^56^ 2018; Guttmann et al,^57^ 2020
Vision screening in schools by contract screeners	3-5	0.810	β	Unpublished KVTP data from Nishimura et al,^42^ 2019
Well-child visit^a^	3-5	0.840	β	Guttmann et al,^57^ 2020
Adherence to treatment for amblyopia or amblyopia risk factor	3-5	0.75	β	Pradeep et al,^58^ 2014; Tailor et al,^59^ 2016
Receipt of social assistance	3-5	0.04	Normal	Maytree,^60^ 2018; Statistics Canada,^61^ 2017
Successful treatment of untreated amblyopia	3	0.89	Log normal	PEDIG data: Holmes et al,^44^ 2003; Cotter et al,^45^ 2006; PEDIG,^46^ 2002; Wallace et al,^47^ 2013; Repka et al,^48^ 2004; Repka et al,^49^ 2008; Scheiman et al,^50^ 2005; Repka et al,^62^ 2005
Successful treatment of untreated amblyopia	4	0.76	Log normal	PEDIG data: Holmes et al,^44^ 2003; Cotter et al,^45^ 2006; PEDIG,^46^ 2002; Wallace et al,^47^ 2013; Repka et al,^48^ 2004; Repka et al,^49^ 2008; Scheiman et al,^50^ 2005; Repka et al,^62^ 2005
Successful treatment of untreated amblyopia	5	0.65	Log normal	PEDIG data: Holmes et al,^44^ 2003; Cotter et al,^45^ 2006; PEDIG,^46^ 2002; Wallace et al,^47^ 2013; Repka et al,^48^ 2004; Repka et al,^49^ 2008; Scheiman et al,^50^ 2005; Repka et al,^62^ 2005
Primary care screening in well-child visits (reference strategy)				
Referral to optometrist after positive screening test	3	0.66	β	Hered and Wood,^63^ 2013
Referral to optometrist after positive screening test	4	0.91	β	Hered and Wood,^63^ 2013
Referral to optometrist after positive screening test	5	0.86	β	Hered and Wood,^63^ 2013
Referral to optometrist after inconclusive screening test	3	0.59	β	Hered and Wood,^63^ 2013
Referral to optometrist after inconclusive screening test	4	0.59	β	Hered and Wood,^63^ 2013
Referral to optometrist after inconclusive screening test	5	0.50	β	Hered and Wood,^63^ 2013
Adherence to referral to optometrist after positive screening test	3	0.63	β	Hered and Wood,^63^ 2013
Adherence to referral to optometrist after positive screening test	4	0.62	β	Hered and Wood,^63^ 2013
Adherence to referral to optometrist after positive screening test	5	0.42	β	Hered and Wood,^63^ 2013
Adherence to referral to optometrist after inconclusive screening test	3	0.34	β	Hered and Wood,^63^ 2013
Adherence to referral to optometrist after inconclusive screening test	4	0.33	β	Hered and Wood,^63^ 2013
Adherence to referral to optometrist after inconclusive screening test	5	0.56	β	Hered and Wood,^63^ 2013
Inconclusive screening test if child has untreated amblyopia	3-5	0.05	β	Unpublished KVTP data from Nishimura et al,^42^ 2019
Inconclusive screening test if child has untreated amblyopia risk factors	3-5	0.03	β	Unpublished KVTP data from Nishimura et al,^42^ 2019
Inconclusive screening test if child has healthy vision or treated amblyopia	3-5	0.02	β	Unpublished KVTP data from Nishimura et al,^42^ 2019
School screening strategy				
Referral to optometrist after positive screening test	5	1.00	NA	Unpublished KVTP data from Nishimura et al,^42^ 2019
Referral to optometrist after inconclusive screening test	5	1.00	NA	Unpublished KVTP data from Nishimura et al,^42^ 2019
Adherence to referral to optometrist after positive screening test	5	0.66	β	Unpublished KVTP data from Nishimura et al,^42^ 2019
Adherence to referral to optometrist after inconclusive screening test	5	0.66	β	Unpublished KVTP data from Nishimura et al,^42^ 2019
Inconclusive screening test if child has untreated amblyopia	5	0.30	β	Unpublished KVTP data from Nishimura et al,^42^ 2019
Inconclusive screening test if child has untreated amblyopia risk factors	5	0.12	β	Unpublished KVTP data from Nishimura et al,^42^ 2019
Inconclusive screening test if child has healthy vision or treated amblyopia	5	0.05	β	Unpublished KVTP data from Nishimura et al,^42^ 2019
**Transition probabilities every year**				
Any health state to death	3	0.00014	NA	Statistics Canada,^43^ 2019
Any health state to death	4	0.00011	NA	Statistics Canada,^43^ 2019
Any health state to death	5	0.00009	NA	Statistics Canada,^43^ 2019
Any health state to death	6	0.00008	NA	Statistics Canada,^43^ 2019
Any health state to death	7	0.00008	NA	Statistics Canada,^43^ 2019
Any health state to death	8	0.00007	NA	Statistics Canada,^43^ 2019
Any health state to death	9	0.00008	NA	Statistics Canada,^43^ 2019
Any health state to death	10	0.00008	NA	Statistics Canada,^43^ 2019
Any health state to death	11	0.00009	NA	Statistics Canada,^43^ 2019
Any health state to death	12	0.0001	NA	Statistics Canada,^43^ 2019
Any health state to death	13	0.00013	NA	Statistics Canada,^43^ 2019
Any health state to death	14	0.00016	NA	Statistics Canada,^43^ 2019
Any health state to death	15	0.00021	NA	Statistics Canada,^43^ 2019
Any health state to death	16	0.00027	NA	Statistics Canada,^43^ 2019
Any health state to death	17	0.00033	NA	Statistics Canada,^43^ 2019
Any health state to death	18	0.00039	NA	Statistics Canada,^43^ 2019
Healthy vision to amblyopia risk factor	3-5	0.033	NA	Donnelly et al,^64^ 2005
Untreated amblyopia to healthy vision (VA ≥20/25)	3-5	0.472	NA	PEDIG data: Holmes et al,^44^ 2003; Cotter et al,^45^ 2006; PEDIG,^46^ 2002; Wallace et al,^47^ 2013; Repka et al,^48^ 2004; Repka et al,^49^ 2008; Scheiman et al,^50^ 2005; Repka et al,^62^ 2005
Untreated amblyopia to treated amblyopia (VA <20/25)	3-5	0.528	NA	PEDIG data: Holmes et al,^44^ 2003; Cotter et al,^45^ 2006; PEDIG,^46^ 2002; Wallace et al,^47^ 2013; Repka et al,^48^ 2004; Repka et al,^49^ 2008; Scheiman et al,^50^ 2005; Repka et al,^62^ 2005
Untreated amblyopia to vision loss in nonamblyopic eye	5-15	0.00004	Log normal	Rahi et al,^65^ 2002
Untreated amblyopia to vision loss in nonamblyopic eye	16-18	0.00005	Log normal	Rahi et al,^65^ 2002
Untreated amblyopia risk factor to healthy vision (VA ≥20/25)	3-5	1.00	NA	Investigator assumption
Untreated amblyopia risk factor to untreated amblyopia	3-5	0.32	β	Unpublished KVTP data from Nishimura et al,^42^ 2019
**Accuracy of screening**				
Primary care screening in well-child visits (reference strategy)				
Sensitivity of screening test to detect untreated amblyopia	3-5	0.659	β	Unpublished KVTP data from Nishimura et al,^42^ 2019
Sensitivity of screening test to detect untreated amblyopia risk factor	3-5	0.586	β	Unpublished KVTP data from Nishimura et al,^42^ 2019
Specificity of screening test	3-5	0.398	β	Unpublished KVTP data from Nishimura et al,^42^ 2019
School screening strategy				
Sensitivity of screening test to detect untreated amblyopia	5	0.867	β	Unpublished KVTP data from Nishimura et al,^42^ 2019
Sensitivity of screening test to detect untreated amblyopia risk factor	5	0.882	β	Unpublished KVTP data from Nishimura et al,^42^ 2019
Specificity of screening test	5	0.121	β	Unpublished KVTP data from Nishimura et al,^42^ 2019
Optometric examination strategy				
Sensitivity of eye exam to detect untreated amblyopia, amblyopia risk factor, or treated amblyopia	3-5	0.95	NA	Gandjour et al,^37^ 2003
**Utility estimates**				
Healthy vision	3-5	1.00	NA	Carlton et al,^33^ 2008; König and Barry,^35^ 2004
Untreated amblyopia	3-5	0.96	NA	van de Graaf et al,^52^ 2010; Membreno et al,^53^ 2002
Untreated amblyopia risk factor	3-5	0.96	NA	van de Graaf et al,^52^ 2010; Membreno et al,^53^ 2002
Vision loss in nonamblyopic eye	3-5	0.93	β	van de Graaf et al,^51^ 2016
Treated amblyopia	3-5	0.99	NA	van de Graaf et al,^52^ 2010; Membreno et al,^53^ 2002

Abbreviations: KVTP, Kindergarten Vision Testing Program; NA, not applicable; PEDIG, Pediatric Eye Investigator Group; VA, visual acuity.

^a^ Weighted mean of the probability of visits and screenings by pediatricians and family physicians.

### Outcomes

Outcomes were measured as incremental quality-adjusted life-years (QALYs). Health utilities for each health state were derived from published and unpublished data on adults because of the lack of relevant data on children younger than 6 years with amblyopia or amblyopia risk factors (eMethods 6 in Supplement 1).^51,52,53^ The utility of amblyopia and amblyopia risk factors was assumed to be the same because both conditions present with the same symptoms. In addition, previous economic evaluations have not distinguished between amblyopia and amblyopia risk factors as separate health states.^12,36,37,67,68^ QALYs were calculated by multiplying utility weights for a particular health state by the duration of time spent in that health state and summed over the time horizon.^69^ Health utilities are listed in Table 1.

### Costs

Direct costs to the Ontario government, including the health care and social services sectors, were identified and estimated for each vision testing strategy. Cost items for the health care sector included consultations with and visits to primary care physicians (ie, family physicians and pediatricians) and optometrists and services provided by public health nurses and contract screeners. Unit prices for these health care sector costs were sourced from the Ontario Schedule of Benefits and Fees,^70,71^ and labor market information was obtained from the Department of Employment and Social Development of the Canadian government.^72^ Cost items in the social service sector included prescription glasses for children with vision impairment who receive social assistance. These social service costs were sourced from the Vision Care Fee Schedule of the Ministry of Community and Social Services.^73^ The cost of patches was not included in the analysis because it would have been a direct cost to families and not the Ontario government.

In each of the alternative strategies, the cost of vision screening (ie, wages and salary of screening personnel and public health nurses who trained the screeners) was estimated for all children tested. Costs of visits to the optometrist were estimated for all children in the optometric examination strategy and only for children who adhered to a referral in the school screening and primary care screening strategies. Costs were expressed in Canadian dollars (CAD; mean exchange rate in 2019: CAD $1.3268 = US $1.00 [converted values may not be exact because they were rounded to the hundredths]). Costs from earlier years were inflated to 2019 CAD and USD using the health care component of the Consumer Price Index.^74^ Table 2 summarizes the resource use, unit costs, and data sources^15,16,33,53,57,70,71,72,73,75,76,77,78,79,80^ used in the model. Valuation of key costs is described in eMethods 7 in Supplement 1.

**Table 2.** Resource Use, Valuation, and Costs in the Microsimulation Model

Event	Unit cost/patient, CAD (USD)^a^	Cost frequency	Data source
Well-child visit^b^	41.34 (31.16)	Annual	Canadian Paediatric Society,^16^ 2009; OMHLTC,^71^ 2019; Guttmann et al,^57^ 2020; Asare et al,^75^ 2021
Vision screening by primary care physicians^b^^,^^c^	11.50 (8.67)	Annual	Guttmann et al,^57^ 2020; Asare et al,^75^ 2021
Vision screening by contract screeners	10.00 (7.54)	Annual	Nishimura et al,^76^ 2020
Optometrist visits and consultations	42.50 (32.03) (diagnostic examination); 25.15 (18.96) (follow-up examinations)	Annual	American Optometric Association,^15^ 2018; Carlton et al,^33^ 2008; Membreno et al,^53^ 2002; OMHLTC,^70^ 2009; American Optometric Association,^77^ 1994; American Optometric Association,^78^ 1997
Training of volunteer screeners by a public health nurse ($41.75 CAD [$31.47] per h)^d^	0.01 (0.01)	Annual	DESD,^72^ 2018; Ontario Nurses’ Association,^79^ 2020; Statistics Canada,^80^ 2008
Prescription glasses (frames, lenses, and case) for social assistance recipients (1 set every 3 y)	120.70 (90.97)	Every 3 y	OMCSS,^73^ 2015

Abbreviations: CAD, Canadian dollars; DESD, Department of Employment and Social Development; OMCSS, Ontario Ministry of Community and Social Services; OMHLTC, Ontario Ministry of Health and Long-Term Care; USD, US dollars.

^a^ Costs are in 2019 dollars (mean exchange rate in 2019: CAD $1.3268 = US $1.00). Converted values may not be exact due to rounding.

^b^ The costs of well-child visits and vision screening by primary care physicians were added in the reference screening strategy (primary care).

^c^ Weighted mean of the proportion of well-child visits and screenings conducted by pediatricians and family physicians.

^d^ Based on National Occupation Classification code 3012 (registered nurses and registered psychiatric nurses).

### Statistical Analysis

#### Cost-effectiveness Analysis

The probabilistic model was constructed and analyzed using TreeAge Pro, health care version 2022 (TreeAge Software).^81^ The results of the probabilistic analysis were reported as mean total costs and mean total QALYs per child for each strategy over 15 years. Mean incremental costs (the difference in total costs associated with implementation of a strategy) and mean incremental QALYs (the difference in benefits associated with implementation of a strategy) were also reported for each comparator strategy (school screening or optometric examinations) relative to the reference strategy (primary care screening). We calculated 95% CIs for point estimates, incremental costs, and QALYs. A 1.5% per year discount rate was applied to costs and health benefits incurred beyond 1 year in the reference case analysis.^82^ When data were available, specified distributions were assigned to model inputs, and values were randomly drawn (eMethods 1 in Supplement 1). The justification for the choice of key model inputs is available in eAppendix 4 and eMethods 3 to 8 in Supplement 1.

An estimate of the incremental cost per QALY gained per child associated with each alternative strategy (school screening or optometric examinations) relative to the reference strategy (primary care screening) was calculated and summarized in an incremental cost-effectiveness ratio (ICER) when possible (eMethods 2 in Supplement 1). The ICER is the additional cost required to achieve an additional QALY beyond what the reference strategy would provide at a willingness-to-pay (WTP) threshold of CAD $50 000 (US $37 690) per QALY gained. The probability that a strategy was cost-effective was determined as the proportion of iterations under a CAD $50 000 (US $37 690) per QALY WTP threshold and summarized in cost-effectiveness acceptability curves.

**Figure 1.**
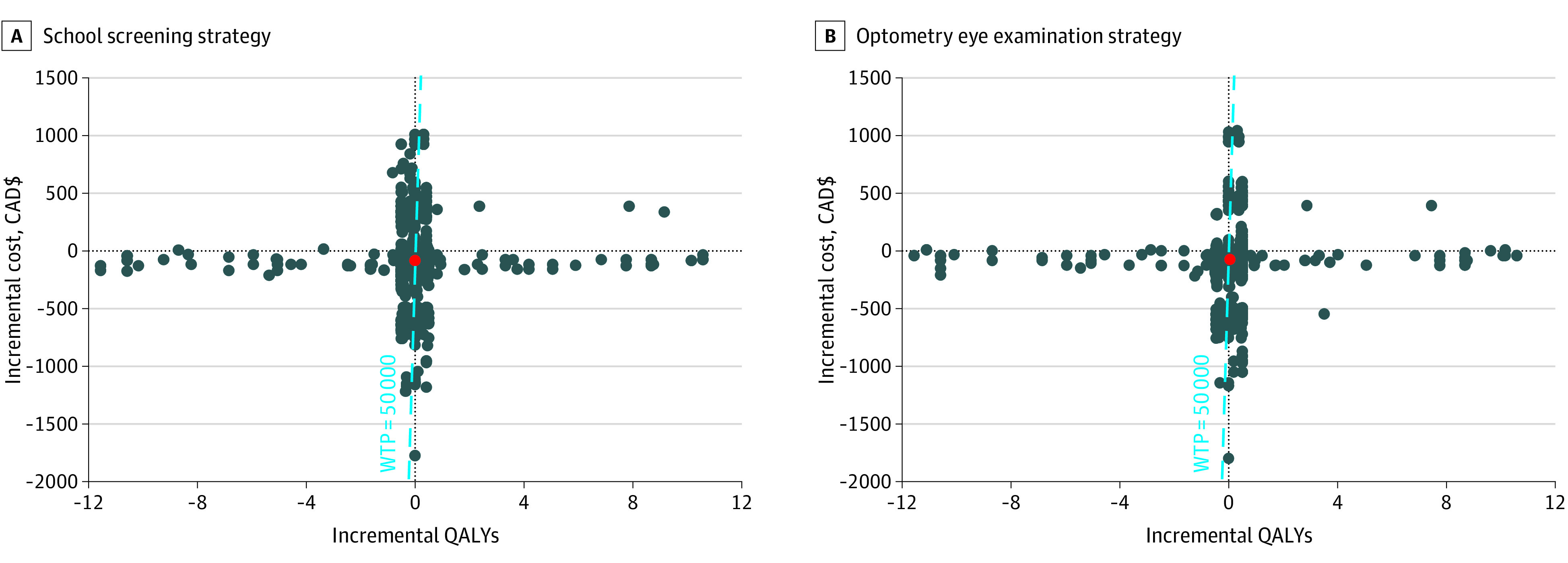
Cost-effectiveness of School Screening and Optometric Examinations vs Vision Screening in Primary Care From the Public Sector Perspective Incremental cost-effectiveness ratios for each of the 25 000 iterations simulated in the probabilistic analysis. The dashed blue diagonal line represents the willingness-to-pay (WTP) threshold of CAD $50 000 (US $37 690) per quality-adjusted life-year (QALY) gained. The dots to the right of the blue dashed line represent cost-effectiveness. Incremental costs are reported in 2019 CAD (mean exchange rate in 2019: CAD $1.3268 = US $1.00).

In the presence of uncertainty, equivalent costs or outcomes between strategies were adjusted to favor the reference strategy to prevent the introduction of bias. When mean incremental QALYs were greater than 0 and mean incremental costs were $0 or greater, iterations were reported as displayed in the northeast quadrant of the cost-effectiveness plane. When mean incremental QALYs were 0 or less and mean incremental costs were $0 or greater, iterations were reported as displayed in the northwest quadrant of the cost-effectiveness plane. When mean incremental QALYs were 0 or less and mean incremental costs were less than $0, iterations were reported as displayed in the southwest quadrant of the cost-effectiveness plane. When mean incremental QALYs were greater than 0 and mean incremental costs were less than $0, iterations were reported as displayed in the southeast quadrant of the cost-effectiveness plane (Figure 1). In addition, a correction factor of +0.0001 was applied to incremental costs, and a correction factor of −0.0001 was applied to the incremental QALYS associated with each iteration in the alternative strategies compared with the reference strategy to account for ICERs that were on the y-axis.^83^

#### Uncertainty and Sensitivity Analysis

The consequences of a range of possible values of key parameters for the ICER were tested in a probabilistic sensitivity analysis. Select parameters, including the cost of prescription glasses and vision screening tests (primary care screening strategy), the sensitivity of optometric examinations, the probability of adhering to treatment, and the utility of untreated amblyopia, were varied one at a time using a specified plausible range of values informed by 95% CIs, data from the literature, or expert opinion (eMethods 8 in Supplement 1) while keeping all other parameters in the model constant. The model parameters and the respective ranges of values tested in the sensitivity analyses are reported in eAppendix 5 and eTable 2 in Supplement 1.

## Results

### Reference Case Analysis

The mean total costs and QALYs per child for each strategy and for the school screening and optometric examination strategies compared with the standard primary care screening strategy over 15 years (discounted at 1.5% from the Ontario government perspective) are shown in Table 3. Compared with the primary care screening strategy, the school screening and optometric examination strategies were generally less costly and had more health benefits. The incremental difference in cost was a savings per child of CAD $84.09 (95% CI, CAD $82.22-$85.95) (US $63.38 [95% CI, US $61.97-$64.78]) for school screening and a savings per child of CAD $74.47 (95% CI, CAD $72.90-$76.03) (US $56.13 [95% CI, $54.95-$57.30]) for optometric examinations. The alternative screening strategy that yielded the largest gain in QALYs compared with the primary care screening strategy was optometric examinations, producing mean QALYs of 0.0508 (95% CI, 0.0455-0.0561) per child (approximately 19 days).

**Table 3.** Mean and Incremental Costs, Mean and Incremental Quality-Adjusted Life-Years, and Incremental Cost-effectiveness Ratios^a^

Strategy	Mean cost per child (95% CI), CAD/USD^b^	Incremental cost per child (95% CI), CAD/USD^b^	Mean QALY per child (95% CI)	Incremental QALY per child (95% CI)	ICER
Primary care screening	147.85 (146.39 to 149.31)/111.43 (110.33 to 112.53)	NA	13.3865 (13.3812 to 13.3918)	NA	NA
School screening	63.76 (62.09 to 65.43)/48.06 (46.80 to 49.32)	−84.09 (−85.95 to −82.22)/−63.38 (−64.78 to −61.97)	13.3869 (13.3817 to 13.3921)	0.0004 (−0.0047 to 0.0055)	Dominant
Optometric examination	73.38 (72.10 to 74.67)/55.31 (54.34 to 56.28)	−74.47 (−76.03 to −72.90)/−56.13 (−57.30 to −54.95)	13.4373 (13.4323 to 13.4422)	0.0508 (0.0455 to 0.0561)	Dominant

Abbreviations: CAD, Canadian dollar; NA, not applicable (reference strategy); QALY, quality-adjusted life-year; USD, US dollar.

^a^ For 25 000 children simulated from the public sector perspective in the reference case probabilistic analysis.

^b^ Costs are in 2019 dollars (mean exchange rate in 2019: CAD $1.3268 = US $1.00). Converted values may not be exact due to rounding.

The reference case ICER results from the probabilistic analysis are illustrated in a scatter plot for each alternative strategy compared with the primary care screening strategy (Figure 1). A total of 73% of iterations comparing optometric examinations with primary care screening and 78% of iterations comparing school screening with primary care screening were on the y-axis, suggesting no change to QALYs. Applying rules in favor of the reference strategy, the school screening and optometric examination strategies compared with the primary care screening strategy were less costly and had more health benefits (Table 3) in 8% and 14% of iterations, respectively (Figure 1).

### Sensitivity Analysis

**Figure 2.**
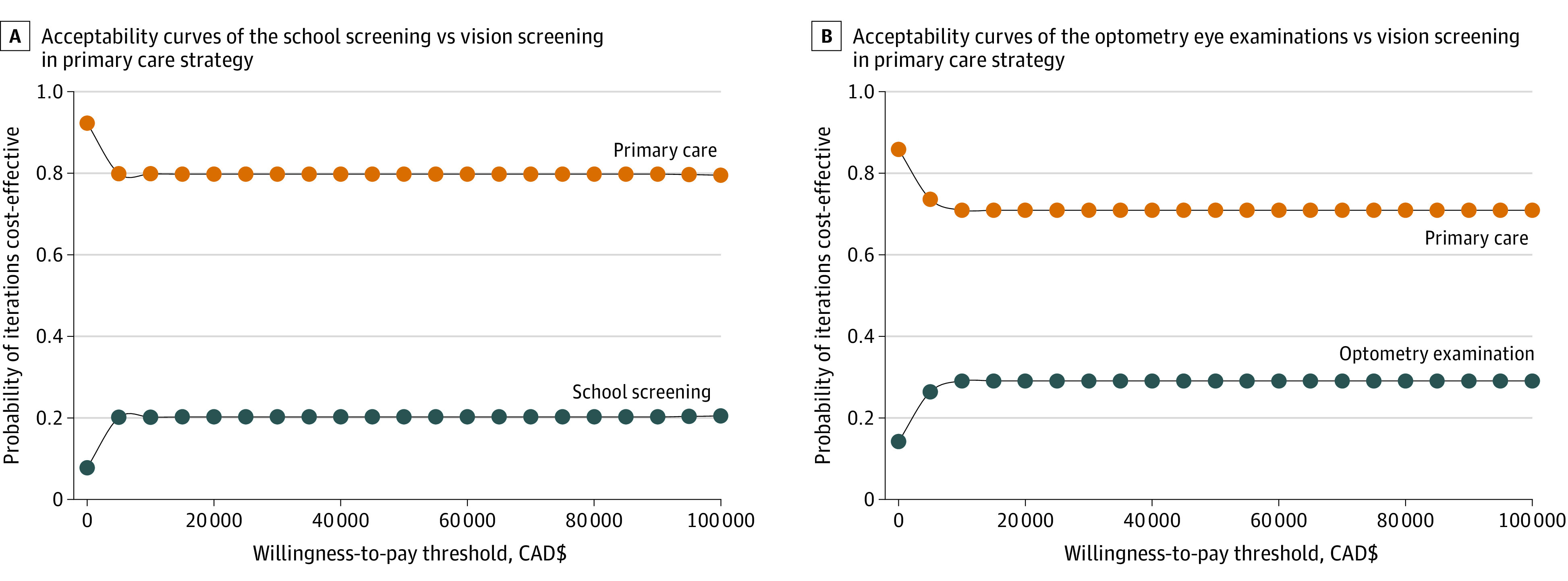
Acceptability Curves of School Screening and Optometric Examinations vs Vision Screening in Primary Care From the Public Sector Perspective Acceptability curves from the reference case analysis. The orange dotted line represents the reference strategy (primary care screening), and the blue dotted line represents the alternative strategy (school screening or optometric examination). Each dot represents the probability of either alternative strategy being cost-effective at willingness-to-pay thresholds of CAD $0 to $100 000 (US $0 to $75 380) per quality-adjusted life-year from the public sector perspective analysis. Costs are reported in 2019 CAD (mean exchange rate in 2019: CAD $1.3268 = US $1.00).

Varying each of these inputs did not produce meaningful changes to the threshold and ICER for the comparison of school screening with primary care screening. The results of the probabilistic analysis for all tested inputs are shown in eTable 2 in Supplement 1. Comparing optometric examinations with primary care screening, if the cost of vision screening was CAD $11.50 (US $8.67), the ICER would be CAD $77.95 (US $58.75) per QALY gained. The cost-effectiveness acceptability curves revealed that at a WTP threshold of CAD $50 000 (US $37 690) per QALY gained, the standard primary care screening strategy was the best policy option for the detection and treatment of amblyopia and amblyopia risk factors for all children aged 3 to 5 years enrolled in public elementary schools in Toronto, Ontario (Figure 2).

## Discussion

In this economic evaluation, we found that the school screening and optometric examination strategies were each less costly and had more health benefits than the primary care screening strategy. However, fewer than 30% of iterations in the school screening and optometric examination strategies were cost-effective relative to the primary care screening strategy at a WTP threshold of CAD $50 000 (US $37 690) per QALY gained. Therefore, reference case results suggested that school screening and optometric examinations were not cost-effective relative to primary care screening for the detection of amblyopia and amblyopia risk factors. These findings were initially unexpected because both school screening and optometric examinations use more sensitive tests to detect amblyopia and amblyopia risk factors, have a higher rate of adherence to referrals, and present fewer opportunities for children to be screened than primary care. However, because there were no added health benefits for testing children with healthy vision, who formed most of the cohort (74%). The mean health benefit obtained per child and the disutility associated with amblyopia and amblyopia risk factors in the reference case may not justify the resources consumed in school screening and optometric examinations.

Few published economic evaluations^33,34,35,36,37,38,67,84,85,86^ have focused on vision testing programs to detect amblyopia or amblyopia risk factors in children. The lack of reliable health state utility data in children with untreated amblyopia has led to the adoption of healthy utility values ranging from 0.83 to 1.00^33,34,35,36,53^ (eMethods 6 in Supplement 1). Previous studies^34,35,36^ that assumed a disutility associated with untreated amblyopia and/or amblyopia risk factors reported vision testing for children aged 3 to 5 years as cost-effective compared with no screening or standard care at a WTP threshold of CAD $50 000 (US $37 690) per QALY gained. The ICER in previous studies^34,35,36,87^ (converted to 2019 values) ranged between CAD $9429 (US $7107; 7397 Deutsch Mark [DM]) and CAD $40 654 (US $30 641; DM 22 083) per QALY gained over a lifetime horizon and CAD $1178 (US $888; DM 924) per additional case detected. The results of these previous studies^34,35,36,87^ are not generalizable to Ontario because of the lack of a true standard care strategy (with reported costs and health benefits); differences in budgeting, organization, and funding of health care; and differences in school systems in other jurisdictions compared with Ontario.

### Strengths and Limitations

This study has several strengths, including its use of a reference strategy with reported costs and final outcomes in terms of QALYs. Most studies^33,34,35,36,37,38,67,86,88,89^ on this topic have used intermediate clinical outcomes, such as the cost per additional case detected, and either lacked a reference strategy or assumed a no screening or standard care strategy with 0 costs and health benefits. Another strength is the use of patient-level data, which assured the derivation of estimates in the school screening strategy that were reflective of the target population. Patient-level data were collected by the Kindergarten Vision Testing Program in field studies involving more than 700 children aged 4 to 5 years who were screened by trained contract screeners in Toronto, Canada, using tools recommended by the Ontario Ministry of Health.^42^ The findings from our current study are likely generalizable to other jurisdictions with health care systems similar to Ontario’s, including other Canadian provinces and territories, the UK, Australia, and others.

This study also has limitations. The opportunity cost of vision screening may have been overestimated. Most physician services within provincial public health care plans in Ontario are reimbursed using a fee-for-service payment scheme that approximates the opportunity cost of the physician’s time. However, well-child visits consist of a package of bundled services, which includes other preventive services with a single fee code.^70,71^ For this reason, the full cost of well-child visits was used in the analysis for the primary care screening strategy even though vision screening comprises only an estimated 25% of the total duration of a typical well-child visit.^90^ A sensitivity analysis using hourly wage rates for family physicians and pediatricians to calculate a weighted mean for the cost of vision screening in well-child visits (eTable 2 in Supplement 1) revealed no substantial changes in the main findings derived in the reference case analysis.^91^

Another limitation was the use of an adult utility estimate of untreated amblyopia for children. Published utility estimates specific to children younger than 6 years with untreated amblyopia or amblyopia risk factors are lacking. Therefore, an adult utility of 0.96 from the literature^53,92^ was assumed (eMethods 6 in Supplement 1). To account for uncertainty in this estimate, the utility of 0.96 was tested probabilistically with a range of 0.83 to 1.00 to account for the range of utility values for untreated amblyopia adopted in previous economic evaluations.^34,35,36^ When the utility of untreated amblyopia was 1.00, school screening was less costly and had less benefit relative to primary care screening, while optometric examinations remained dominant for the range tested.

Resource use and quality of life impacts associated with vision testing and subsequent treatment in school screening and optometric examinations compared with primary care screening were not fully captured in this analysis because of its limited time horizon of 15 years. A lifetime time horizon was not possible due to lack of long-term data for resource use and QALY losses. Therefore, our study results do not provide a complete picture of the cost-effectiveness of the alternative strategies to standard care for appropriate decision-making for resource allocation.

Some assumptions may have had substantial implications for the results of the analysis. Children who were not diagnosed with amblyopia or an amblyopia risk factor had lasting vision impairment that would not resolve on its own. This assumption resulted in more favorable results for optometric examinations relative to primary care screening in the reference case analysis because of the higher sensitivity of testing in optometric examinations. Other assumptions include the adjustment of incremental costs and outcomes of 0 to favor the reference strategy, which reversed the results of the analysis because most of the population (74%) had normal (or healthy) vision. Another assumption was that children had no other form of vision impairment, such as refractive errors not large enough to cause amblyopia but nevertheless believed to negatively impact quality of life by causing reading and learning difficulties.^93,94,95,96,97,98^ In addition, we did not consider social issues, such as bullying, low self-esteem and self-image, strained family relationships, and other problems, which have been associated with undergoing patching treatment for amblyopia among children.^99,100,101,102^ The omission of other factors associated with vision impairment and social issues would produce an underestimation of the benefits of the testing strategies.

## Conclusions

This economic evaluation found that in a universally funded single-payer health care system, alternative strategies (ie, universal school screening and optometric examinations) were not cost-effective relative to vision screening that routinely occurs in primary care for detecting amblyopia and its risk factors because most children in the cohort did not have these disorders. The mean added health benefits associated with the alternative strategies compared with the primary care screening strategy would not warrant the resources used.

## Notes

Supplement 1. **eAppendix 1.** Model Structure **eFigure 1.** Detailed Model Describing Events and Transitions for All Vision Testing Strategies **eAppendix 2.** Referral Criteria Provided by the Ontario Ministry of Health for Children Screened in the Public Health School Vision Screening Program **eTable 1.** Definitions of Suspected Amblyopia, Amblyopia Risk Factors, and Inconclusive Screenings That Informed Referrals to an Optometrist in the Ontario Ministry of Health Vision Screening Program **eAppendix 3.** Additional Methods **eFigure 2.** Markov Model for the Vision Testing Cost-effectiveness Model **eMethods 1.** Assigning Distributions to Variables **eMethods 2.** Reporting Incremental Cost-effectiveness Ratios **eAppendix 4.** Computing Key Variables **eMethods 3.** Epidemiological Data **eMethods 4.** Transition Probabilities **eMethods 5.** Probabilities **eMethods 6.** Measuring Effects **eMethods 7.** Quantifying Costs **eMethods 8.** Variables Used in the 1-Way Probabilistic Sensitivity Analyses **eAppendix 5.** Additional Results **eTable 2.** Model Parameters Tested in the 1-Way Probabilistic Sensitivity Analyses and Consequences of the Lower and Upper Limit of the Range of Each Model Input for the Incremental Cost-effectiveness Ratios of School Screening and Optometric Examinations vs Primary Care From the Public Sector Payer Perspective
eReferences

Supplement 2.
Data Sharing Statement
