# Supplementary material for: Cost-effectiveness of Universal School- and Community-Based Vision Testing Strategies to Detect Amblyopia in Children in Ontario, Canada
Source: JAMA Netw Open. 2023 Jan 4;6(1):e2249384. doi: 10.1001/jamanetworkopen.2022.49384 (PMC9857467; doi:10.1001/jamanetworkopen.2022.49384)
Supplement: Supplement 1. — eAppendix 1. Model Structure eFigure 1. Detailed Model Describing Events and Transitions for All Vision Testing Strategies eAppendix 2. Referral Criteria Provided by the Ontario Ministry of Health for Children Screened in the Public Health School Vision Screening Program eTable 1. Definitions of Suspected Amblyopia, Amblyopia Risk Factors, and Inconclusive Screenings That Informed Referrals to an Optometrist in the Ontario Ministry of Health Vision Screening Program eAppendix 3. Additional Methods eFigure 2. Markov Model for the Vision Testing Cost-effectiveness Model eMethods 1. Assigning Distributions to Variables eMethods 2. Reporting Incremental Cost-effectiveness Ratios eAppendix 4. Computing Key Variables eMethods 3. Epidemiological Data eMethods 4. Transition Probabilities eMethods 5. Probabilities eMethods 6. Measuring Effects eMethods 7. Quantifying Costs eMethods 8. Variables Used in the 1-Way Probabilistic Sensitivity Analyses eAppendix 5. Additional Results eTable 2. Model Parameters Tested in the 1-Way Probabilistic Sensitivity Analyses and Consequences of the Lower and Upper Limit of the Range of Each Model Input for the Incremental Cost-effectiveness Ratios of School Screening and Optometric Examinations vs Primary Care From the Public Sector Payer Perspective eReferences [file jamanetwopen-e2249384-s001.pdf]

# Supplemental Online Content

Asare AO, Maurer D, Wong AMF, Saunders N, Ungar WJ. Cost-effectiveness of universal school- and community-based vision testing strategies to detect amblyopia in children in Ontario, Canada. *JAMA Netw Open*. 2023;6(1):e2249384. doi:10.1001/jamanetworkopen.2022.49384

**eAppendix 1.** Model Structure

**eFigure 1.** Detailed Model Describing Events and Transitions for All Vision Testing Strategies

**eAppendix 2.** Referral Criteria Provided by the Ontario Ministry of Health for Children Screened in the Public Health School Vision Screening Program

**eTable 1.** Definitions of Suspected Amblyopia, Amblyopia Risk Factors, and Inconclusive Screenings That Informed Referrals to an Optometrist in the Ontario Ministry of Health Vision Screening Program

**eAppendix 3.** Additional Methods

**eFigure 2.** Markov Model for the Vision Testing Cost-effectiveness Model

**eMethods 1.** Assigning Distributions to Variables

**eMethods 2.** Reporting Incremental Cost-effectiveness Ratios

**eAppendix 4.** Computing Key Variables

**eMethods 3.** Epidemiological Data

**eMethods 4.** Transition Probabilities

**eMethods 5.** Probabilities

**eMethods 6.** Measuring Effects

**eMethods 7.** Quantifying Costs

**eMethods 8.** Variables Used in the 1-Way Probabilistic Sensitivity Analyses

**eAppendix 5.** Additional Results

**eTable 2.** Model Parameters Tested in the 1-Way Probabilistic Sensitivity Analyses and Consequences of the Lower and Upper Limit of the Range of Each Model Input for the Incremental Cost-effectiveness Ratios of School Screening and Optometric Examinations vs Primary Care From the Public Sector Payer Perspective

**eReferences**

This supplemental material has been provided by the authors to give readers additional information about their work.

© 2023 Asare AO et al. *JAMA Network Open*.

The information included in this supplemental document provides additional details of the methodology adopted and results for the study that could not be included in the manuscript due to space limitations. It summarizes the model structure, definitions of target conditions that informed referrals in the universal school screening strategy, additional results from the public sector payer perspective, and a description of how key model inputs were derived which. Further information is available upon request from the corresponding author (wendy.ungar@sickkids.ca).

## **eAppendix 1.** Model Structure

### **eFigure 1.** Detailed Model Describing Events and Transitions for All Vision Testing Strategies

a. Possible Pathways of an Individual Simulated in the Healthy Vision Health State in the Reference Strategy of the Vision Testing Cost-effectiveness Model

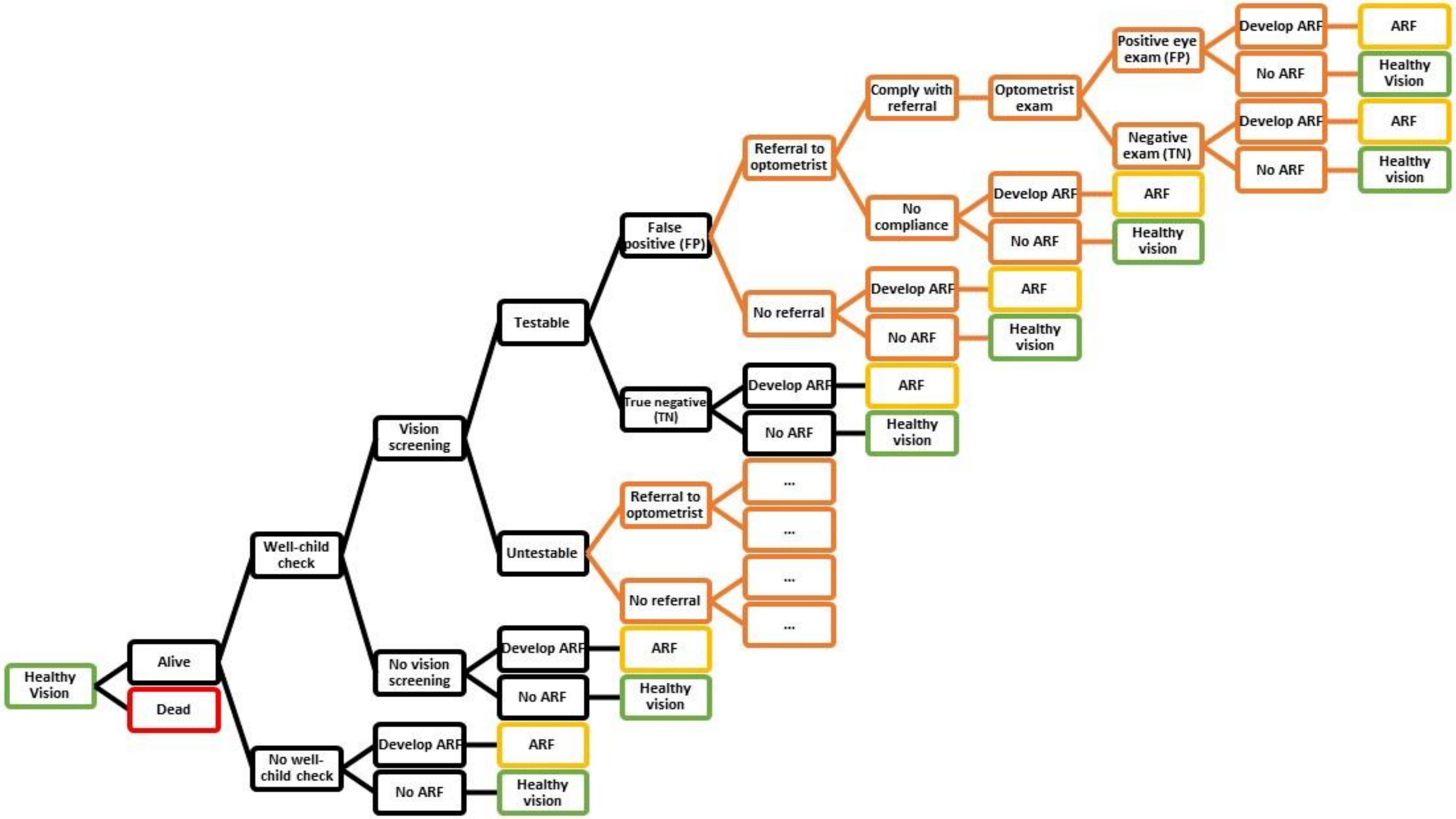


All coloured boxes except orange boxes indicate the different health states in the model. Orange boxes represent the portion of the pathway that is repeated (i.e., clones). Three dots (…) indicate where repetition of the earlier orange pathway has occurred. Costs of unnecessary referrals were factored into the analysis as a result of false positive screening results.

© 2023 Asare AO et al. *JAMA Network Open*.

b. Possible Pathways of an Individual Simulated in the Untreated Amblyopia Risk Factor Health State in the Reference Strategy of the Vision Testing Cost-effectiveness Model

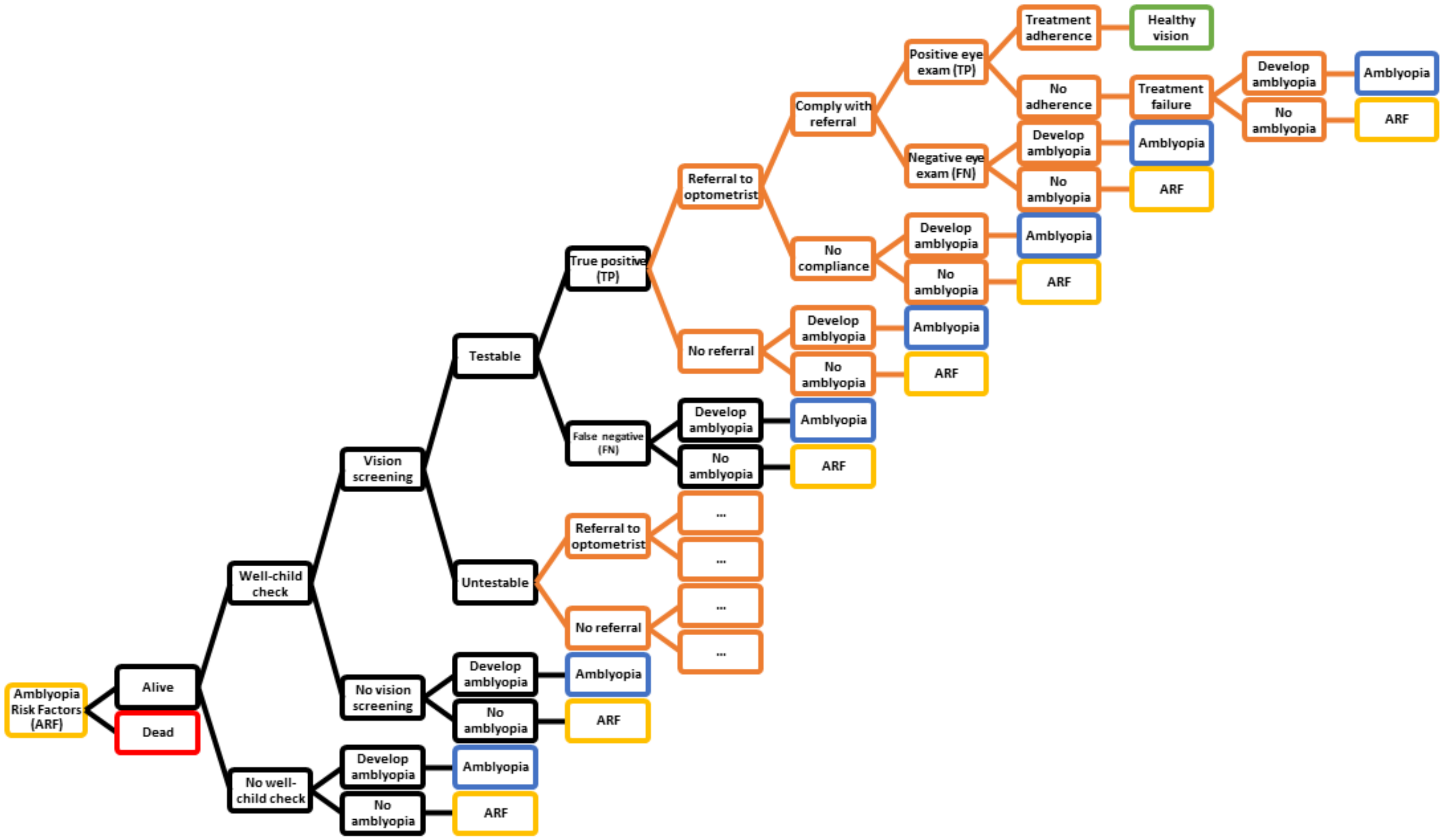


All coloured boxes except orange boxes indicate the different health states in the model. Orange boxes represent the portion of the pathway that is repeated (i.e., clones). Three dots (…) indicate where repetition of the earlier orange pathway has occurred.

© 2023 Asare AO et al. *JAMA Network Open*.

c. Possible Pathways of an Individual Simulated in the Untreated Amblyopia Health State in the Reference Strategy of the Vision Testing Cost-effectiveness Model

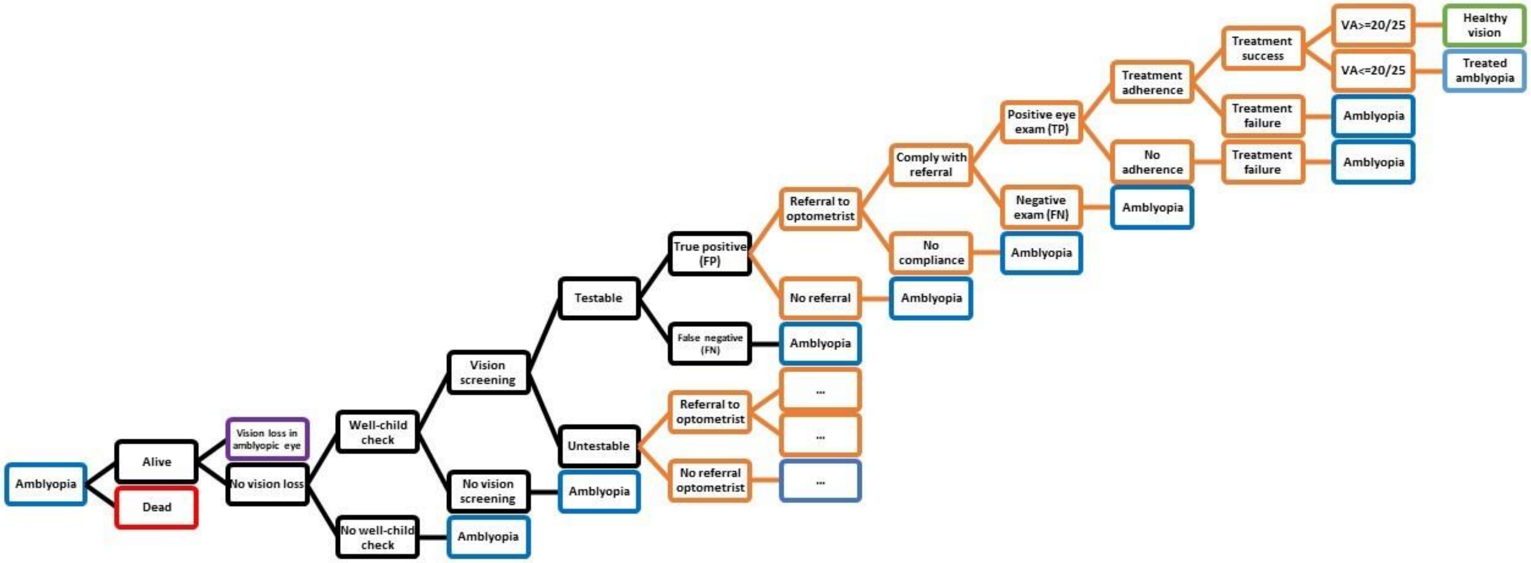


All coloured boxes except orange boxes indicate the different health states in the model. Orange boxes represent the portion of the pathway that is repeated (i.e., clones). Three dots (…) indicate where repetition of the earlier orange pathway has occurred.

© 2023 Asare AO et al. *JAMA Network Open*.

d. Possible Pathways of an Individual Simulated in the Treated Amblyopia Health State in the Reference Strategy of the Vision Testing Cost-effectiveness Model

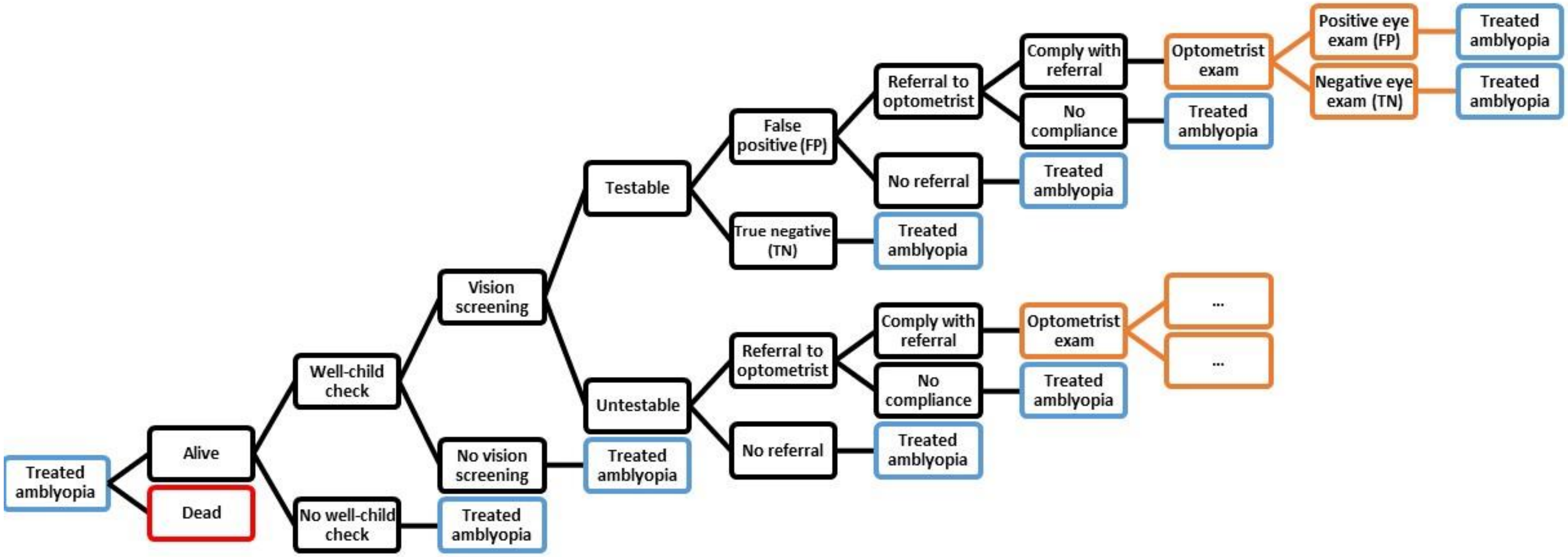


All coloured boxes except orange boxes indicate the different health states in the model. Orange boxes represent the portion of the pathway that is repeated (i.e., clones). Three dots (…) indicate where repetition of the earlier orange pathway has occurred. Costs of unnecessary referrals were factored into the analysis as a result of false positive screening results.

© 2023 Asare AO et al. *JAMA Network Open*.

e. Pathways from the Vision Loss in Non-Amblyopic Eye and Dead Health State in the Reference Strategy of the Vision Testing Cost-effectiveness Model

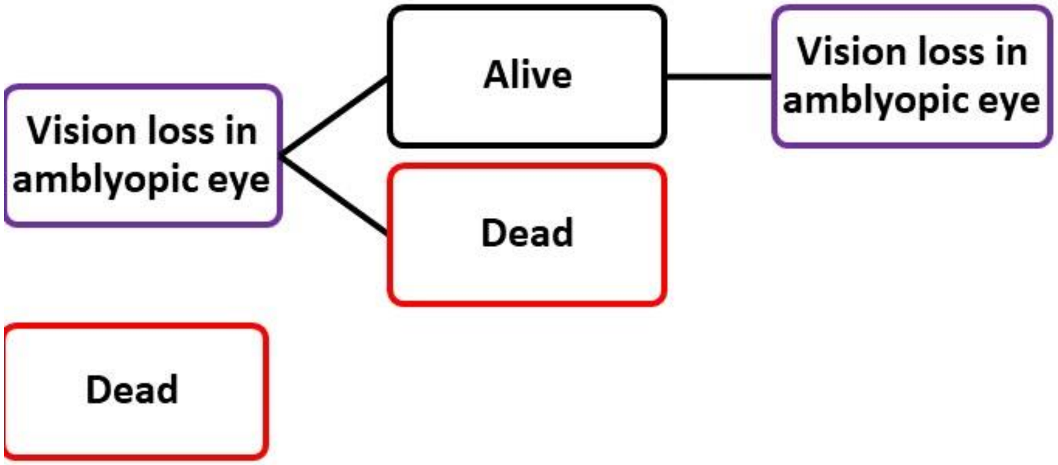


All coloured boxes except orange boxes indicate the different health states in the model. Orange boxes represent the portion of the pathway that is repeated (i.e., clones). Three dots (…) indicate where repetition of the earlier orange pathway has occurred.

© 2023 Asare AO et al. *JAMA Network Open*.

**eAppendix 2.** Referral Criteria Provided by the Ontario Ministry of Health for Children Screened in the Public Health School Vision Screening Program

**eTable 1.** Definitions of suspected amblyopia, amblyopia risk factors, and inconclusive screenings that informed referrals to an optometrist in the Ontario Ministry of Health vision screening program

| Type of Vision Impairment | Definition/Thresholds for Referral |
|---|---|
| Unilateral amblyopia | worse than 20/32 visual acuity line in one or both eyes |
| Amblyopia risk factors: | |
| • Reduced stereoacuity | unable to identify ≥2 shapes at 60 arcsec |
| Refractive errors: | |
| • Hyperopia | > 1.0D |
| • Myopia | < -1.5D |
| • Astigmatism | <-0.75D or >0D |
| • Anisometropia | > 1.5D |
| Inconclusive: | |
| • Child unable or unwilling to do the test | |

D = diopter

© 2023 Asare AO et al. *JAMA Network Open*.

**eAppendix 3.** Additional Methods

**eFigure 2.** State Diagram of the Markov Model for Vision Testing to Detect Amblyopia and Amblyopia Risk Factors

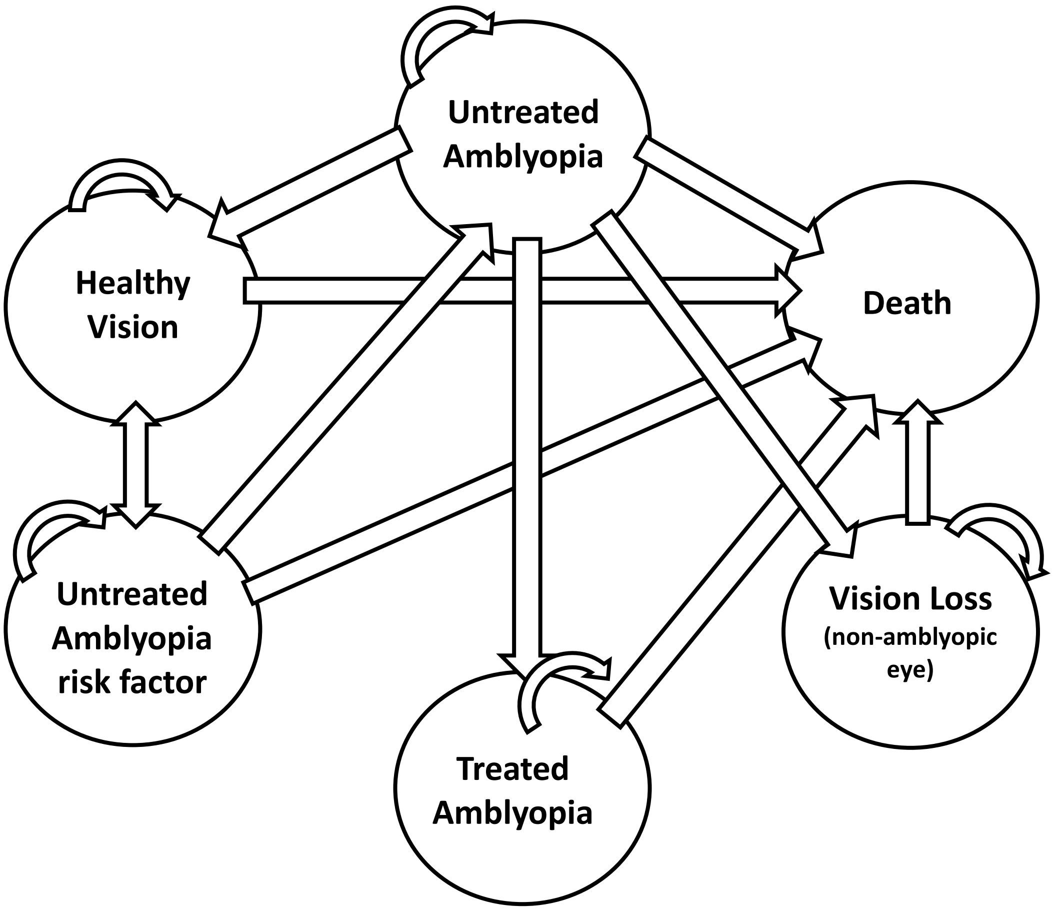


© 2023 Asare AO et al. *JAMA Network Open*.

**eMethods 1.** Assigning Distributions to Variables

Utilities were assigned beta distributions because they cover continuous variables between 0 to 1. Similarly, beta distributions were assigned when probabilities were estimated from proportions, and lognormal distributions when estimated from logistic regression equations. A normal distribution was assigned when the probability was estimated from a large sample size (>100,000). A Poisson distribution was assigned for discrete, positive counts such as the number of optometry visits.(Drummond, Sculpher, Claxton, Stoddart, & Torrance, 2015). The table below provides details of the distributions used in the model. Parameters listed in Table 1 in the manuscript but not in the table below were not assigned distributions because there was not enough data available in the published/unpublished literature to determine an appropriate distribution. Therefore, they were not varied in the probabilistic model.

| **Model Parameter** | **Distribution used in simulation** | **Data Source** |
|---|---|---|
| **Epidemiological data** | | |
| Prevalence of untreated amblyopia at age 3 yrs | Beta (92, 1487) | Tarczy-Hornoch et al. 2011 |
| Prevalence of untreated amblyopia risk factors at age 3 yrs | Beta (140; 552) | KVTP data: Nishimura et al, 2019 |
| **Probability of events** | | |
| Vision screening in schools by contract screeners | Beta (5477,1308) | KVTP data: Nishimura et al, 2019 |
| Well-child visit[a] | Beta (765 072, 145) | Guttman et al. 2020 |
| Adherence to treatment for amblyopia or amblyopia risk factor | Beta (31, 10) | Pradeep et al. 2014, Tailor et al. 2016 |
| Receipt of social assistance at 3-5 yrs | Normal (0.04, 0.01) | Maytree 2018, Statistics Canada 2017 |
| Successful treatment of untreated amblyopia at 3 yrs | Lognormal (-3.24, 0.89) | PEDIG data |
| Successful treatment of untreated amblyopia at 4 yrs | Lognormal (-2.66, 0.76) | PEDIG data |
| Successful treatment of untreated amblyopia at 5 yrs | Lognormal (−2.46, 0.65) | PEDIG data |
| Referral to optometrist after: | | |
| Positive screening test at age 3 yrs | Beta (19, 10) | Hered and Wood, 2013 |
| Positive screening test at age 4 yrs | Beta (32, 3) | Hered and Wood, 2013 |
| Positive screening test at age 5 yrs | Beta (25, 4) | Hered and Wood, 2013 |
| Inconclusive screening test at age 3 yrs | Beta (138, 95) | Hered and Wood, 2013 |
| Inconclusive screening test at age 4 yrs | Beta (138, 95) | Hered and Wood, 2013 |
| Inconclusive screening test at age 5 yrs | Beta (47, 48) | Hered and Wood, 2013 |
| Adherence to referral to optometrist after: | | |
| Positive screening test at age 3 yrs | Beta (10, 6) | Hered and Wood, 2013 |
| Positive screening test at age 4 yrs | Beta (18, 11) | Hered and Wood, 2013 |
| Positive screening test at age 5 yrs | Beta (8, 11) | Hered and Wood, 2013 |
| Inconclusive screening test at age 3 yrs | Beta (39, 76) | Hered and Wood, 2013 |
| Inconclusive screening test at age 4 yrs | Beta (13, 27) | Hered and Wood, 2013 |
| Inconclusive screening test at age 5 yrs | Beta (5, 4) | Hered and Wood, 2013 |
| Inconclusive screening test if patient has: | | |
| Untreated amblyopia at 3-5 years | Beta (2, 41) | KVTP data: Nishimura et al, 2019 |
| Untreated amblyopia risk factors at 3-5 years | Beta (4,140) | KVTP data: Nishimura et al, 2019 |
| Healthy vision or treated amblyopia at 3-5 yrs | Beta (12,511) | KVTP data: Nishimura et al, 2019 |
| **School Screening Strategy** | | |
| Adherence to referral to optometrist after: | | |
| Positive screening test at 5 yrs | Beta (363, 188) | KVTP data: Nishimura et al, 2019 |
| Inconclusive screening test at 5 yrs | Beta (363, 188) | KVTP data: Nishimura et al, 2019 |
| Inconclusive screening test if one has: | | |
| Untreated amblyopia at 5 yrs | Beta (13, 30) | KVTP data: Nishimura et al, 2019 |
| Untreated amblyopia risk factors at 5 yrs | Beta (17; 127) | KVTP data: Nishimura et al, 2019 |
| Healthy vision or treated amblyopia at 5 yrs | Beta (25, 498) | KVTP data: Nishimura et al, 2019 |
| **Transition Probabilities** | | |
| In the following year, patient: | | |

© 2023 Asare AO et al. *JAMA Network Open*.

| | | |
|---|---|---|
| with amblyopia risk factor developing untreated amblyopia at 3-5 yrs | Beta (18, 38) | KVTP data: Nishimura et al, 2019 |
| Developing vision loss in non amblyopic eye at age 5-15yrs | Lognormal (-10.13, 0.46) | Rahi et al. 2002 |
| Developing vision loss in non amblyopic eye at age 16-18yrs | Lognormal (-9.90, 0.46) | Rahi et al. 2002 |
| **Accuracy of screening** | | |
| *Primary care screening in well-child visits (reference strategy)* | | |
| Sensitivity of vision test to detect: | | |
| Untreated amblyopia at 3-5 yrs | Beta (27, 14) | KVTP data: Nishimura et al, 2019 |
| Untreated amblyopia risk factor at 3-5 yrs | Beta (82, 58) | KVTP data: Nishimura et al, 2019 |
| Specificity of vision test at 3-5 yrs | (Beta 72, 109) | KVTP data: Nishimura et al, 2019 |
| *School screening strategy* | | |
| Sensitivity of vision test to detect: | | |
| Untreated amblyopia at 5 yrs | Beta (26, 4) | KVTP data: Nishimura et al, 2019 |
| Untreated amblyopia risk factor at 5 yrs | Beta (112, 15) | KVTP data: Nishimura et al, 2019 |
| Specificity of vision test at 5 yrs | Beta (19, 138) | KVTP data: Nishimura et al, 2019 |
| **Utility estimates** | | |
| Vision loss in nonamblyopic eye at 3-5yrs | Beta (7, 0.57) | van de Graaf et al,2016 |

Parameters for a beta distribution are alpha (a) and beta (b) values derived from binomial data. These are represented in parenthesis after the distribution. For binomial data: a = r, b =(n-r) for r events out of n individuals. For instance, we found in the study by Tarczy et al 2011 that 92 out of 1487 3-year old's from the sample were determined to have amblyopia in one eye. Therefore, alpha was the number of events experienced (92), and beta value = total number of patients (1579) minus (92 events)= 1,487. For Lognormal distributions, numbers in the brackets are (μ, δ) where, μ - mean of logs, δ-standard deviation of logs', [a]weighted average of the probability of visits and screenings by pediatricians and family physicians. Abbreviations: KVTP – Kindergarten Vision Testing Program, VA- visual acuity, PEDIG – Pediatric Eye Investigator Group.

© 2023 Asare AO et al. *JAMA Network Open*.

**eMethods 2.** Reporting Incremental Cost-effectiveness Ratios (ICERs)

An ICER was not reported for any comparator strategy that was dominated absolutely or by extension (i.e. less costly but more effective, or more costly and less effective than the reference strategy).

© 2023 Asare AO et al. *JAMA Network Open*.

## **eAppendix 4.** Computing Key Variables

A description of the derivation of key variables used in the model is described in the following sections.

## **eMethods 3.** Epidemiological Data

The following epidemiological data points listed in table 1 are explained below.

**Prevalence of untreated amblyopia risk factors = 0.20**

The prevalence of amblyopia risk factors for children at age 3 is a ‘starter’ probability that informs how children in the cohort of 25,000 simulated children are distributed across the health states in cycle 0 (i.e., first year in the model) at age 3 years in each strategy. It was derived from unpublished patient-level data from the Kindergarten Vision Testing Program (University of Toronto/McMaster University, Canada). 140 out of 692 children screened in Ontario, Canada aged 4-5 years were determined to have an amblyopia risk factor at baseline. Children were screened with visual acuity charts, stereoacuity charts and an autorefractor.

**Prevalence of untreated amblyopia = 0.058 (at age 3)**

The prevalence of untreated amblyopia was determined from a paper by Tarczy-Hornoch et al. in which 92 out of 1487 3-year old’s from an ethnically diverse population-based sample from California and Maryland were determined to have amblyopia in one eye (i.e., an interocular distance in VA≥2 lines with ≤20/32 in the worse eye).(Tarczy-Hornoch et al., 2011)

© 2023 Asare AO et al. *JAMA Network Open*.

## **eMethods 4.** Transition Probabilities

The following transition probabilities listed in table 1 are explained below.

**Healthy vision → untreated amblyopia risk factor (0.03):**

This parameter (like all other transition probabilities in the model) is the probability that a child with healthy vision will transition (or develop) to another health state (in this case, untreated amblyopia risk factor) in subsequent cycles of the model (cycle 1-15, or year ≥2). The probability of developing untreated amblyopia risk factor in subsequent years was based on a retrospective study by Donnelley et al. in which 198 children with normal vision in a population of 1,582 developed significant amblyopia risk factors (strabismus, and refractive error causing amblyopia) over a period of 4 years.(Donnelly, Stewart, & Hollinger, 2005) Converting to an annual incident rate, 198/(1582*4) = 0.03

**Successful treatment of untreated amblyopia:** i) **Untreated amblyopia → healthy/normal vision (0.472), ii) Untreated amblyopia → treated amblyopia (0.528)**

The probability of a child 3-5 years of age with untreated amblyopia developing a visual acuity (VA) ≥ 20/25 (normal/healthy vision) – 0.472 after successful treatment was derived from two studies by the Pediatric Eye Disease Investigator Group (PEDIG) led by Beck et al. and Repka et al.(Beck, 2003; Repka et al., 2003) VA in the amblyopic eye and sound eye was measured after 6 months of patching for 6 hours a day in children 3-7 years of age with moderate and severe amblyopia. Across both studies, 91/281 children had VA ≥ 20/25 (0.32). Converting this rate to an annual probability = 0.472. Subsequently, the probability of a child 3-5 years of age with untreated amblyopia developing a VA < 20/25 = 1-0.472 = 0.528

**Untreated amblyopia risk factor → healthy vision (1)**

Amblyopia risk factors are disorders of the eye, specifically, strabismus (“eye turns”), anisometropia (significant differences in the magnitude of refractive errors between the eyes), anisocoria (Unequal size of the pupils) and media opacities resulting from congenital cataract or ptosis, that put a child at risk for the development of amblyopia (S. P. Donahue et al., 2013). Impaired vision (i.e. poor visual acuity) can be restored to normal (or near normal) if amblyopia risk factors are detected and treated early. (Brown, Brown, Sharma, & Busbee, 2003; Sean P. Donahue, 2006; Grossman et al., 2017; Langelaan et al., 2007; D. K. Wallace et al., 2017) For these reasons, we assumed in the model that all children with an amblyopia risk factor that were treated at an early age and who adhered to treatment prescribed regained normal (or healthy) vision. Children that did not adhere to treatment remained with an amblyopia risk factor in a subsequent cycle of the model (refer to eFigure 1b in supplementary materials).

**Untreated amblyopia risk factor → untreated amblyopia (0.32)**

This parameter was derived from unpublished patient-level data from the Kindergarten Vision Testing Program (University of Toronto/Mcmaster University, Canada). It represented the proportion of children aged ~ 5 years diagnosed with an amblyopia risk factor by an optometrist in Ontario, Canada. 18 out of 56 children examined developed amblyopia ~ 1.5 years after they were diagnosed with an amblyopia risk factor. We assumed that children that did not adhere to treatment remained with an amblyopia risk factor in a subsequent cycle of the model (refer to eFigure 1b)

© 2023 Asare AO et al. *JAMA Network Open*.

## **eMethods 5.** Probabilities

Unable to Test

A child was considered 'unable to test' if they were unable or unwilling to complete the test for any of the testing tools used in each strategy, or if there was equipment malfunction. It was assumed that all children were able to complete testing in the gold-standard OE strategy. The probability that a child would be unable to complete a screening test in the USS and PC strategies were derived from unpublished field data from the Kindergarten Vision Testing Program of 710 four and 5 year old children enrolled in kindergartens in Ontario (Nishimura, Wong, Cohen, Thorpe, & Maurer, 2019). The rates were determined as a function of the health state i.e., amblyopia, healthy vision, and amblyopia risk factor. Children with amblyopia or amblyopia risk factor were more likely to be unable to complete a test (Maguire, 2007). Although American Academy of Pediatrics guidelines recommend rescreening untestable children before referral, children were assumed in this model to be tested only once, therefore the probability of being untestable reflects a single instance of untestability. The effectiveness of a rescreening strategy for untestable children was not considered. If children were re-screened this would impact only the PC strategy, as all children were referred in the SS strategy if they were unable, and all children were assumed to complete a test in the OE strategy (i.e., no unables). Therefore, the result of a re-screening strategy would result in a less favourable (lower) ICER than the reference case analysis for the alternative strategies (OE and SS) relative to PC.

Testing Accuracy: Sensitivity and Specificity of Testing

The sensitivity of tests to detect amblyopia, amblyopia risk factor and specificity of tests to determine healthy vision was derived from unpublished data from the Kindergarten Vision Testing Program for the SS and PC strategies for the combination of tools used in each strategy (Nishimura et al., 2019). The proportion of children that were unable to complete a test were excluded from the derivation of test accuracy for each vision-testing strategy because they were treated separately in the Markov model (refer pathways in appendix A – eFigure 1). Thereafter, the specific decisions regarding referrals for each testing strategy were applied to the data (i.e., testing results for each child) to determine children that failed screening (Table S1 above). The data was then compared to gold-standard vision examination results by an optometrist for each participant in the trial, to determine the true-positive rate (sensitivity) and true-negative rate (specificity). Sensitivity was derived for individuals with amblyopia or amblyopia risk factor, while specificity was derived for those with healthy vision or treated amblyopia. In the SS strategy, testing accuracy was determined using decisions for referral by the Ministry of Health (Table S1 above). Because of a lack of data on the specific referral criteria used in the PC strategy, the same referral criterion was assumed as that used by the Ministry of Health for visual acuity testing only. In the OE strategy test accuracy was assumed to be 95% sensitive and 95% specific, in line with a previous study by Gandjour et al. (Gandjour, Schlichtherle, Neugebauer, Russmann, & Lauterbach, 2003).

*Mandatory comprehensive eye exams*

Relevant data on the probability that a child had an OE could not be found in the literature. Therefore, we assumed that this probability would be the same as the reported proportion of children that received mandatory vaccinations in Ontario in 2013 and tested it in a one-way probabilistic analysis (Walkinshaw, 2011)

Background Mortality

This data was derived from Statistics Canada and was the probability of death between age $x$ and $x+1$ for both sexes in Ontario (Statistics Canada, 2016-18). That is, the probability that a person of age $x$ dies before reaching age $x+n$. Information with regards to the margin of error associated with the death probability at each age came from the same source (Statistics Canada, 2016-18).

© 2023 Asare AO et al. *JAMA Network Open*.

Vision screening as Part of Well-Child Checks by Primary Care Physicians

The probability of vision screening as part of well-child checks was estimated using data from a survey in Ontario by Le et al. data. The survey from 2013 conducted on family physicians/general practitioners (n=1,000) and pediatricians (n=1,000) in Ontario reported that of those that responded to the survey (719, 24%), only 65% of family physicians and general practitioners (65% of 449 =292), and 52% of pediatricians (52% of 270=140) conducted vision screening at every WCC (Le et al., 2018). The response rate of the survey was low which limits generalizability of the results. Unlike the results of the survey by Le et. al, other studies have shown evidence of increased uptake of preventive screening such as enhanced well child visits and immunizations in Ontario by paediatricians compared to family physicians (Campitelli, Inoue, Calzavara, Kwong, & Guttmann, 2012; Guttmann et al., 2020). Guttmann et al. and Campitelli et al. determined a higher odds of receiving an enhanced well-child visit (28% higher odds) and full immunizations (85% higher odds) respectively amongst children cared for by a pediatrician compared with patients enrolled in a family physician led primary care model. To account for uncertainty in this data point from Le et. al used in the reference case analysis, the parameter was tested probabilistically and in a one-way probabilistic sensitivity analysis. We sampled the variable (i.e., probability of vision screening as part of well-child checks) probabilistically from a beta distribution informed by the probability of vision screening by a family physician from Le et al. We had no reason to believe that the shape of the distribution would be different for family physicians compared to paediatricians.

Well-Child Visits

There was limited data on the probability of well-child visits conducted by primary care providers in children 3 to 5 years of age in Ontario. The base value of 84% used in the reference case analysis represented the proportion of children aged 17 to 24 months receiving well-child visits in Ontario in which 54% of children (n=494,108) had an 18-month well-child visit and 65% of those that did not get an 18-month well-child visit (n=269,664) got a regular well-child visit (Guttmann et al., 2020)

© 2023 Asare AO et al. *JAMA Network Open*.

## **eMethods 6.** Measuring Effects

*Utility of untreated amblyopia, amblyopia risk factors, treated amblyopia, and vision loss in the non-amblyopic eye*
There is no validated technique for measuring health utility in young children, therefore there was no relevant data on the utility associated with the target conditions in children under 6 years of age (Carlton, Karnon, Czoski-Murray, Smith, & Marr, 2008; Griebsch, Coast, & Brown, 2005; König & Barry, 2004; Rein et al., 2012). For this reason, the utility for the target conditions was estimated from the literature. (Membreno, Brown, Brown, Sharma, & Beauchamp, 2002; van de Graaf, van Kempen-du Saar, Looman, & Simonsz, 2010). The utility of untreated amblyopia (0.96) was obtained from a study using the time trade-off method from a sample of 135 adults (mean age 41 years) with persistent amblyopia and/or strabismus who were treated with occlusion therapy by the same orthoptist 30-35 years prior. (van de Graaf et al., 2010)

Membreno et al. determined the difference in utility before and immediately after treatment for amblyopia to be0.03 utility units per year (Membreno et al., 2002). Therefore, for this current study, a treatment gain of 0.03 utility units was subsequently applied to the utility of 0.96 for untreated amblyopia. Therefore, the utility of treated amblyopia was assumed to be 0.99 (i.e., 0.96 + 0.0).

The utility of vision loss in the non-amblyopic eye (0.93) was derived from a sample of adults (mean age 70 years) with recent decrease of vision in their better eye to a visual acuity of less than 20/40 in both eyes. These patients had persistent amblyopia and age-related macular degeneration.(van de Graaf, Despriet, Klaver, & Simonsz, 2016)

Assuming adult utilities for children may overestimate the disutility associated with amblyopia or amblyopia risk factors. This is because the lack of binocular vision could impact career choices and hobbies in adults, which would not be applicable to young children. Therefore, a range of utilities of 0.83 to 1 for amblyopia and amblyopia risk factors based on reference values used in previous economic evaluation studies, was assumed in the one-way probabilistic analysis for this study (Carlton et al., 2008; König & Barry, 2004; Membreno et al., 2002; Rein et al., 2012). Carlton et al. assumed no disutility associated with unilateral vision impairment in the reference case analysis and a range of 0.98 to 1 was tested in a one-way probabilistic analysis (Carlton et al., 2008). Konig et al. used a utility of 0.96 in the reference case based on a utility study on adult populations with unilateral vision impairment by Brown et al and applied an arbitrary range of 0.92 to 1.00 in their sensitivity analysis to reflect the lack of evidence of disutility due to unilateral vision impairment (König & Barry, 2004). The difference in the mean utility of unilateral and bilateral good vision in a study by Brown et al. was 0.08. Membreno et al. assumed a mean utility value of 0.83 corresponding to vision of 20/20 in one eye and 20/80 in the second eye (untreated amblyopia) using a database of 35 consecutive patients with this vision. (Membreno et al., 2002). Finally, Rein et al. assumed an arbitrary mean disutility of 0.01 per year of unresolved amblyopic monocular impairment and varied this from 0.035 to 0.040 in a sensitivity analysis (Rein et al., 2012).

© 2023 Asare AO et al. *JAMA Network Open*.

## **eMethods 7.** Quantifying Costs

*Well-child checks by family physicians and pediatricians*
These unit prices were derived from the Ontario Schedule of Benefits (Ministry of Health and Long Term Care, 2016). Well-child checks were determined to be billed as 'periodic health visits' for a child aged 2 to 11 years (K017 for family physicians) which are limited to one per patient per 12-month period (Ministry of Health and Long Term Care, 2016)

*Non-Physician Professional Services*
Optometrist Visits and Consults
The cost of optometrist visits and consults (i.e., comprehensive eye exams and follow up visits) were derived from the Ontario Ministry of Health Schedule of Benefits for Optometry Services (Ministry of Health and Long-Term Care, 2009). That is, 'periodic oculo-visual assessment' (fee code V404) and 'oculo-visual minor assessments' (fee code V402). The periodic oculo-visual assessment is defined in the schedule as an assessment of the eye and vision system for patients aged 19 or less or age 65 or more. The service is limited to one per patient per consecutive 12-month period. Oculo-visual minor assessments are defined in the schedule as subsequent eye exams for patients aged 19 or less by the same optometrist to re-assess a single ocular condition. Diagnosis and treatment for amblyopia was assumed to occur over an average of 21 visits with an optometrist, and at least 19 visits (i.e., assuming death did not occur before the end of treatment). It was assumed that when a child complied with the first visit and diagnosed with amblyopia or amblyopia risk factors, he/she continued to attend all visits, but may have not adhered to the treatment. Children with an amblyopia risk factor were assumed to visit their optometrist every 6 months for treatment for a year, and for 37 weeks for children treated for amblyopia.

Public Health Nurse Services
In the reference case, it was assumed that a public health nurse employed full-time by the Toronto Public Health Unit trained contracted screeners for one full day of training (7 hours) in the SS strategy. A median wage of $36.06/hour was derived using publicly available labour market information for the Toronto region from the Department of Employment and Social Development Canada (ESDC) (Government of Canada, 2018). The range of the wage for public health nurses in Toronto per the same labour market information that informed the choice of upper and lower limits of the range used in the one-way probabilistic analysis was 22.6/hours to 45.47/hour (Government of Canada, 2018). Additional compensation for vacation time and sick leave per the Ontario Nurses' Association collective agreement was included (Conference Board of Canada, 2012). This amounted to an additional 4.8% of the median wage ($1.73). According to The Conference Board of Canada (2012), benefits are 10% of all compensation (wages plus benefits) on a Canada-wide basis (Conference Board of Canada, 2012). As such, per the CADTH costing guidance document, an additional 11% was added onto monetary wages to determine an all-sector, Canada-wide compensation rate for fringe benefits (pensions, supplemental health insurance, life, and disability insurance) (Jacobs, Budden, & Lee, 2016). Therefore, fringe benefits and compensation amounted to ~ 15.8% of the median wage. Taking into consideration fringe benefits and compensation, the total median wage for public health nurses was therefore determined to be $41.75/hr. Therefore a one-time cost of $292.25 for 7 hours of training; which is $0.012 per child per year (i.e., $292.25/25,000 children screened) was determined.(Government of Canada, 2018)"Similarly, the lower range for the one-way probabilistic sensitivity analysis was estimated to be $26.2/hr and the upper range was $52.65 for 7 hours.

*Program Labour*
Contract Screeners from the Gift of Sight and Sound Program
The plan by the Toronto Health Unit is to contract volunteer screeners from the Toronto Foundation for Student Success to coordinate and manage all aspects of the vision screening including data entry. The Toronto Foundation for Student Success charges a flat fee per trial of C$10.

*Direct Costs to Ministry of Community and Social Services*

Prescription Glasses – Ontario Drug Support Program recipients

In the reference case analysis, the cost of a pair of prescription glasses (i.e. frame, lens and case) to the Ministry of Community and Social Services for social assistance beneficiaries through the Ontario Drug Support Program and Ontario Works were estimated using allowable amounts payable (Ontario Ministry of Community and Social Services, 2015). Children were assumed to use basic, plastic single vision lenses with scratch resistance coating, specifically, polycarbonate lenses ($76.50). Frame and case costs were fixed per the vision care fee schedule at $42.40 and $2.00 respectively and assumed to be purchased by all individuals. Therefore, the total cost of prescription glasses, frames and lenses in the reference case was $120.70. The cost of prescription glasses for children receiving social assistance was estimated once every 3 years – in line with the vision care fee schedule.

© 2023 Asare AO et al. *JAMA Network Open*.

## **eMethods 8.** Variables Used in the 1-Way Probabilistic Sensitivity Analyses

The following parameters were tested in the one-way probabilistic analysis because they were expected to have significant impact on study results and/or there was significant uncertainty surrounding the choice of the point estimate. In some cases, the variable was an assumption based on expert opinion, and/or fixed in the reference analysis because of the lack of relevant data to inform the choice of a distribution.

1. **Annual discount rate:** A range of 0% (undiscounted) to 3% per year discount rate will be applied to a one-way probabilistic analysis. This range has been recommended in the CADTH guidelines to reflect twice the recommended base case analysis rate of 1.5% (Canadian Agency for Drugs and Technologies in Health, 2017).
2. **Ministry amounts payable for prescription glasses for ODSP recipients:** The Ministry of Community and Social Services pays a portion of the cost of prescription glasses once every three years depending on the type of lenses and lens coatings selected. Ministry costs for prescription glasses (including a case and frame) could range between C$112.70 to C$265.45 based on a Vision Care Fee Schedule. This range was tested in the one-way probabilistic analysis. The lower limit of the range of the cost of prescription glasses by beneficiaries in the one-way probabilistic analysis was assumed to be the bare minimum of allowable amounts for the single vision lens (i.e. CR39 lenses with scratch resistance coating), while the upper limit of the range was assumed to be the maximum allowable amounts for the single vision lenses with a lens addition [i.e., polycarbonate lenses ($76.50) with photochromic tints ($105) and a high sphere power ($39.75)] which amounted to $144.75. Including the cost of a frame and a case, this amounted to $112.70 for the lower limit, and $265.45 for the upper limit (Ontario Ministry of Community and Social Services, 2015)
3. **Probability of healthy vision at age 3 years**: As an initial distribution of the model which could significantly impacts costs, the probability of healthy vision was tested in the one-way probabilistic analysis. The range of 0.73 and 0.78 was informed by another CUA on vision screening to detect amblyopia in children (Rein et al., 2012).
4. **Probability of adherence to treatment**: In a published review of childhood amblyopia from the UK by Tailor et al, adherence to treatment for amblyopia was reported in two studies (Tailor, Bossi, Greenwood, & Dahlmann-Noor, 2016). In one study by Wallace et al, adherence to refractive adaptation, in addition to occlusion (for a period of 12 weeks or until best visual acuity was reached) was reported to range between 47% to 88%, depending on the duration of occlusion prescribed (6hrs/day vs. 12hrs/day) and the operational measure of compliance/adherence adopted (M. P. Wallace, Stewart, Moseley, Stephens, & Fielder, 2013). Children were in the refractive adaptation phase of treatment for 18 weeks, and the median time spent in the occlusion phase of treatment was 99 days (M. P. Wallace et al., 2013). The other study by Pradeep et al on occlusion alone reported compliance rates over 12 weeks of treatment as 45.2% without educational/motivational intervention material to 80.6% with educational/motivational intervention material (Pradeep et al., 2014). This study looks at refractive adaption in addition to occlusion treatment for amblyopia/amblyopia risk factors for 6 hrs/day. Guidelines from the American Academy of Ophthalmologists (D. K. Wallace et al., 2017) advises the use of a prescribed dose of 6 h for severe amblyopia and 2 h for moderate amblyopia. Clinical expert opinion from the Kindergarten Vision Testing Program indicate that adherence compliance rates could be higher than reported in these studies because health professionals are likely to prescribe fewer hours (2 h) (Maconachie & Gottlob, 2015). Therefore, a range of probability of treatment adherence of 0.50 to 0.93 was adopted and tested in a one-way probabilistic analysis.
5. **Probability of having a mandatory OE:** The rates of vaccinations in Ontario were reported as 84% to 92% of children (Walkinshaw, 2011). This range (0.84 to 0.92) was used in the one-way probabilistic analysis.

© 2023 Asare AO et al. *JAMA Network Open*.

6. **Probability of vision screening as part of a PC**: To account for uncertainty in the data point, the parameter was tested probabilistically and in a one-way probabilistic sensitivity analysis. The range tested in the one-way probabilistic sensitivity analysis was determined using the higher odds (28%) of a well-child screening by paediatricians vs family physicians reported by Guttmann et al (who used linked health administrative and demographic databases of Ontario residents) to inform the upper limit of the range tested (i.e., 0.69). The range used (i.e., 0.59 to 0.69) was further supported by a study by Asare et al. (30% higher odds of a well-child screening by paediatricians vs family physicians) and two American studies by Wall et al and Anzeljc et al. (Anzeljc et al., 2019; Wall et al.). Wall et al conducted a survey on a random sample of 888 American paediatricians and reported that on average, 69% of children aged 3 to 5 years had a visual acuity test as part of well-child visits by their paediatricians (Wall et al.). Anzeljc et al. reported that in the 2016-2017 National Survey of Children's Health 64.9% of children 3 to 6 years old received at least one vision screening nationwide and in the state of Ohio, but the type of care professional providing the test was not specified (Anzeljc et al., 2019).
7. **Sensitivity of tests in OE strategy:** The sensitivity of tests may differ significantly depending on the population for which the testing is done. In the absence of data on possible sensitivity of tests in the population of interests, the range was determined to be ±50% above and below the reference case value (i.e., with an upper ceiling of 1) in the OE.
8. **Cost of vision screening as part of well-child checks:** To account for the overestimation of the opportunity cost of vision screening this, a one-way probabilistic sensitivity analysis was conducted using an alternative calculation to monetize physician's time to approximate the opportunity cost. An hourly wage was derived based on the actual income received by Ontario pediatricians and family physicians in 2019 (i.e., using a combination of fee-for-service and salary payment programs) from a Canadian Institute of Health Information report for 2019 (Canadian Institute for Health Information, 2020). We then assigned a cost of vision screening based on the amount of time spent on vision screening (i.e., 25% of the total duration of a well-child visit) using expert clinical opinion from a pediatrician (N.S) and calculated a weighted average as a more accurate representation of the opportunity cost of vision screening by a pediatrician and family physician.

© 2023 Asare AO et al. *JAMA Network Open*.

**eAppendix 5.** Additional Results

***Results of Sensitivity Analysis from Public Payer Perspective***
Parameters tested in the one-way probabilistic analysis and their respective ranges are described in eTable 2.

**eTable 2.** Model Parameters Tested in the 1-Way Probabilistic Sensitivity Analyses and Consequences of the Lower and Upper Limit of the Range of Each Model Input for the Incremental Cost-effectiveness Ratios (ICERs) of School Screening and Optometric Examinations vs Primary Care From the Public Sector Payer Perspective

(a) School screening vs primary care

| Parameter | Reference Case Value in probabilistic analysis | Distribution | Range Tested in One-Way probabilistic analysis | Impact of Range on ICER of SS vs PC | | | Source of Range |
|---|---|---|---|---|---|---|---|
| | | | | Lower limit | Mean (reference case value) | Upper Limit | |
| Annual discount rate | 0.015 | - | 0.00 to 0.03 | Dominant | Dominant | Dominant | (Canadian Agency for Drugs and Technologies in Health, 2017) |
| Cost of prescription glasses to Ministry | 120.70 | - | 112.7 to 265.45 | Dominant | Dominant | Dominant | (Ontario Ministry of Community and Social Services, 2015) |
| Cost of vision screening as part of well-child checks | 43.14 | - | 11.50 to 43.14 | Dominant | Dominant | Dominant | Report on physician payments in Canada (Canadian Institute for Health Information, 2020), Data from 2010 Ontario Birth Cohort from ICES, Toronto |

© 2023 Asare AO et al. *JAMA Network Open*.

| | | | | | | | |
|---|---|---|---|---|---|---|---|
| | | | | | | | (A. O. Asare, Maurer, Wong, Ungar, & Saunders, 2020), (Guttmann et al., 2020) |
| Sensitivity of comprehensive eye exams | 0.95 | - | 0.48 to 1.00 | - | Dominant | Dominant | Assumption |
| Probability of healthy vision | 0.742 | - | 0.73 to 0.78 | Dominant | Dominant | Dominant | (Rein et al., 2012) |
| Probability of adhering to treatment | 0.75 (31,10) | Beta | 0.50 to 0.93 | - | Dominant | Dominant | (Maconachie & Gottlob, 2015), (M. P. Wallace et al., 2013) |
| Probability of vision screening as part of PC | 0.62* | Beta | 0.59 to 0.69 | Dominant | Dominant | - | (Afua Oteng Asare, Maurer, Wong, Ungar, & Saunders, 2021; Guttmann et al., 2020; Wall et al.) |
| Probability of comprehensive eye exams (OE strategy) | 0.88 | - | 0.84 to 0.92 | Dominant | Dominant | Dominant | (Walkinshaw, 2011) |
| Utility of untreated amblyopia & untreated amblyopia risk factor | 0.96 | - | 0.83 to 1 | Dominant | Dominant | - | (Rein et al., 2012) (König & Barry, 2004) (Carlton et al., 2008) |
| Utility of treated amblyopia | 0.99 | - | 0.83 to 1 | - | Dominant | Dominant | Assumption, Clinical expert opinion (Kindergarten Vision Testing Program) |

© 2023 Asare AO et al. *JAMA Network Open*.

(b) Optometric examinations vs primary care

| Parameter | Reference Case Value in probabilistic analysis | Distribution | Range Tested in One-Way probabilistic analysis | Impact of Range on ICER of OE vs. PC | | | Source of Range |
|---|---|---|---|---|---|---|---|
| | | | | Lower limit | Mean (reference case value) | Upper Limit | |
| Annual discount rate | 0.015 | - | 0.00 to 0.03 | Dominant | Dominant | Dominant | (Canadian Agency for Drugs and Technologies in Health, 2017) |
| Cost of prescription glasses to Ministry | 120.70 | - | 112.7 to 265.45 | Dominant | Dominant | Dominant | (Ontario Ministry of Community and Social Services, 2015) |
| Cost of vision screening as part of well-child checks | 43.14 | - | 11.50 to 43.14 | $77.95 per QALY gained | Dominant | Dominant | Report on physician payments in Canada (Canadian Institute for Health Information, 2020), Data from 2010 Ontario Birth Cohort from ICES, Toronto (A. O. Asare et al., 2020), (Guttmann et al., 2020) |
| Sensitivity of comprehensive eye exams | 0.95 | - | 0.48 to 1.00 | Dominant | Dominant | Dominant | Assumption |
| Probability of healthy vision | 0.742 | - | 0.73 to 0.78 | Dominant | Dominant | Dominant | (Rein et al., 2012) |

© 2023 Asare AO et al. *JAMA Network Open*.

| | | | | | | | |
|---|---|---|---|---|---|---|---|
| Probability of adhering to treatment | 0.75 (31,10) | Beta | 0.50 to 0.93 | Dominant | Dominant | Dominant | (Maconachie & Gottlob, 2015), (M. P. Wallace et al., 2013) |
| Probability of vision screening as part of PC | 0.62* | Beta | 0.59 to 0.69 | Dominant | Dominant | Dominant | (Le et al., 2018), (Guttmann et al., 2020; Wall et al.) |
| Probability of comprehensive eye exams (OE strategy) | 0.88 | - | 0.84 to 0.92 | Dominant | Dominant | Dominant | (Walkinshaw, 2011) |
| Utility of untreated amblyopia | 0.96 | - | 0.83 to 1 | Dominant | Dominant | Dominant | (Rein et al., 2012) (König & Barry, 2004) (Carlton et al., 2008; Membreno et al., 2002) |
| Utility of untreated amblyopia risk factor | 0.96 | - | 0.83 to 1 | Dominant | Dominant | Dominant | (Rein et al., 2012) (König & Barry, 2004) (Carlton et al., 2008) |
| Utility of treated amblyopia | 0.99 | - | 0.83 to 1 | Dominant | Dominant | Dominant | (Membreno et al., 2002) |

Incremental cost-effectiveness ratios (ICERs) were not calculated and reported for dominant or dominated strategies in line with the CHEERS guidelines.(Husereau et al., 2022) The hyphens indicate that a distribution was not assigned to the model inputs in question

© 2023 Asare AO et al. *JAMA Network Open*.

## eReferences


Anzeljc, S., Ziemnik, L., Koscher, S., Klein, W., Bridge, C., & Van Horn, A. (2019). Preschool Vision Screening Collaborative: Successful Uptake of Guidelines in Primary Care. *Pediatr Qual Saf, 4*(6), e241. doi:10.1097/pq9.0000000000000241

Asare, A. O., Maurer, D., Wong, A. M. F., Ungar, W. J., & Saunders, N. (2020). *Material Deprivation and Eye Exams in Young Children in Ontario, Canada: A Population-based Cohort Study.* Paper presented at the Pediatric Academic Societies Meeting 2021, Virtual.

Asare, A. O., Maurer, D., Wong, A. M. F., Ungar, W. J., & Saunders, N. (2021). Socioeconomic Status and Vision Care Services in Ontario, Canada: A Population-Based Cohort Study. *J Pediatr*. doi:https://doi.org/10.1016/j.jpeds.2021.10.020

Beck, R. W. (2003). A Randomized Trial of Prescribed Patching Regimens for Treatment of Severe Amblyopia in Children. *Ophthalmology, 110*(11), 2075-2087. doi:10.1016/j.ophtha.2003.08.001

Brown, M. M., Brown, G. C., Sharma, S., & Busbee, B. (2003). Quality of life associated with visual loss. *Ophthalmology, 110*(6), 1076-1081. doi:10.1016/s0161-6420(03)00254-9

Campitelli, M. A., Inoue, M., Calzavara, A. J., Kwong, J. C., & Guttmann, A. (2012). Low rates of influenza immunization in young children under Ontario's universal influenza immunization program. *Pediatrics, 129*(6), e1421-1430. doi:10.1542/peds.2011-2441

Canadian Institute for Health Information. (2020). *Physicians in Canada, 2019*. Retrieved from Ottawa, ON:

Carlton, J., Karnon, J., Czoski-Murray, C., Smith, K. J., & Marr, J. (2008). The clinical effectiveness and cost-effectiveness of screening programmes for amblyopia and strabismus in children up to the age of 4-5 years: a systematic review and economic evaluation. *Health Technol Assess, 12*(25), iii, xi-194.

Conference Board of Canada. (2012). *Benefits benchmarking 2012*. Retrieved from Ottawa:

Donahue, S. P. (2006). Relationship between anisometropia, patient age, and the development of amblyopia. *Am J Ophthalmol, 142*(1), 132-140. e131. Retrieved from http://www.sciencedirect.com/science/article/pii/S0002939406003357

Donahue, S. P., Arthur, B., Neely, D. E., Arnold, R. W., Silbert, D., Ruben, J. B., & Committee, P. O. S. V. S. (2013). Guidelines for automated preschool vision screening: a 10-year, evidence-based update. *J AAPOS, 17*(1), 4-8. doi:10.1016/j.jaapos.2012.09.012

Donnelly, U. M., Stewart, N. M., & Hollinger, M. (2005). Prevalence and outcomes of childhood visual disorders. *Ophthalmic Epidemiol, 12*(4), 243-250. doi:10.1080/09286580590967772

Drummond, M. F., Sculpher, M. J., Claxton, K., Stoddart, G. L., & Torrance, G. W. (2015). *Methods for the economic evaluation of health care programmes*: Oxford university press.

Gandjour, A., Schlichtherle, S., Neugebauer, A., Russmann, W., & Lauterbach, K. W. (2003). A cost-effectiveness model of screening strategies for amblyopia and risk factors and its application in a german setting. *Optometry & Vision Science, 80*(3), 259-269.

Government of Canada. (2018, Aug 20, 2018). Labour market information. Retrieved from https://www.jobbank.gc.ca/explorecareers


© 2023 Asare AO et al. *JAMA Network Open*.

Griebsch, I., Coast, J., & Brown, J. (2005). Quality-Adjusted Life-Years Lack Quality in Pediatric Care: A Critical Review of Published Cost-Utility Studies in Child Health. *Pediatrics, 115*(5), e600-e614. doi:10.1542/peds.2004-2127

Grossman, D. C., Curry, S. J., Owens, D. K., Barry, M. J., Davidson, K., Doubeni, C. A., . . . Tseng, C. W. (2017). Vision screening in children aged 6 months to 5 years: US preventive services task force recommendation statement. *JAMA - Journal of the American Medical Association, 318*(9), 836-844. doi:10.1001/jama.2017.11260

Guttmann, A., Saunders, N. R., Kumar, M., Gandhi, S., Diong, C., MacCon, K., & Cairney, J. (2020). Implementation of a Physician Incentive Program for 18-Month Developmental Screening in Ontario, Canada. *J Pediatr, 226*, 213-220.e211. doi:10.1016/j.jpeds.2020.03.016

Husereau, D., Drummond, M., Augustovski, F., de Bekker-Grob, E., Briggs, A. H., Carswell, C., . . . Staniszewska, S. (2022). Consolidated Health Economic Evaluation Reporting Standards (CHEERS) 2022 Explanation and Elaboration: A Report of the ISPOR CHEERS II Good Practices Task Force. *Value Health, 25*(1), 10-31. doi:10.1016/j.jval.2021.10.008

König, H.-H., & Barry, J.-C. (2004). Cost-Utility Analysis of Orthoptic Screening in Kindergarten: A Markov Model Based on Data From Germany. *Pediatrics, 113*(2), e95. Retrieved from http://pediatrics.aappublications.org/content/113/2/e95.abstract

Langelaan, M., de Boer, M. R., van Nispen, R. M., Wouters, B., Moll, A. C., & van Rens, G. H. (2007). Impact of visual impairment on quality of life: a comparison with quality of life in the general population and with other chronic conditions. *Ophthalmic Epidemiol, 14*(3), 119-126. doi:10.1080/09286580601139212

Le, T. D., Raashid, R. A., Colpa, L., Noble, J., Ali, A., & Wong, A. (2018). Paediatric vision screening in the primary care setting in Ontario. *Paediatrics & Child Health, 23*(3), e33-e39. doi:10.1093/pch/pxx148

Maconachie, G. D. E., & Gottlob, I. (2015). The challenges of amblyopia treatment. *Biomedical Journal, 38*(6), 510-516. doi:https://doi.org/10.1016/j.bj.2015.06.001

Maguire, M. G. (2007). Children unable to perform screening tests in vision in preschoolers study: proportion with ocular conditions and impact on measures of test accuracy. *Invest Ophthalmol Vis Sci, 48*(1), 83-87. doi:10.1167/iovs.06-0384

Membreno, J. H., Brown, M. M., Brown, G. C., Sharma, S., & Beauchamp, G. R. (2002). A cost-utility analysis of therapy for amblyopia. *Ophthalmology, 109*(12), 2265-2271. Retrieved from https://www.ncbi.nlm.nih.gov/pubmed/12466169

Ministry of Health and Long Term Care. (2016). Schedule of Benefits Physician Services under the Health Insurance Act. Retrieved from http://www.health.gov.on.ca/en/pro/programs/ohip/sob/physserv/physserv_mn.html

Nishimura, M., Wong, A., Cohen, A., Thorpe, K., & Maurer, D. (2019). Choosing appropriate tools and referral criteria for vision screening of children aged 4-5 years in Canada: a quantitative analysis. *BMJ Open, 9*(9), e032138-e032138. doi:10.1136/bmjopen-2019-032138

Ontario Ministry of Community and Social Services. (2015). *Vision Care Fee Schedule.* Retrieved from http://ontario-opticians.com/wp-content/uploads/2015/08/MCSS-Vision-Care-Fee-Schedule-August-2015.pdf

© 2023 Asare AO et al. *JAMA Network Open*.

Pradeep, A., Proudlock, F. A., Awan, M., Bush, G., Collier, J., & Gottlob, I. (2014). An educational intervention to improve adherence to high-dosage patching regimen for amblyopia: a randomised controlled trial. *British Journal of Ophthalmology, 98*(7), 865. doi:10.1136/bjophthalmol-2013-304187

Rein, D. B., Wittenborn, J. S., Zhang, X., Song, M., Saaddine, J. B., & Vision Cost-effectiveness Study, G. (2012). The potential cost-effectiveness of amblyopia screening programs. *J Pediatr Ophthalmol Strabismus, 49*(3), 146-155; quiz 145, 156. doi:10.3928/01913913-20110823-02

Repka, M. X., Beck, R. W., Holmes, J. M., Birch, E. E., Chandler, D. L., Cotter, S. A., . . . Scheiman, M. M. (2003). A randomized trial of patching regimens for treatment of moderate amblyopia in children. *Archives of Ophthalmology, 121*(5), 603-611. doi:10.1001/archopht.121.5.603

Tailor, V., Bossi, M., Greenwood, J. A., & Dahlmann-Noor, A. (2016). Childhood amblyopia: current management and new trends. *Br Med Bull, 119*(1), 75-86. doi:10.1093/bmb/ldw030

Tarczy-Hornoch, K., Varma, R., Cotter, S. A., McKean-Cowdin, R., Lin, J. H., Borchert, M. S., . . . Giordano, L. (2011). Risk factors for decreased visual acuity in preschool children: The multi-ethnic pediatric eye disease and baltimore pediatric eye disease studies. *Ophthalmology, 118*(11), 2262-2273. doi:10.1016/j.ophtha.2011.06.033

van de Graaf, E. S., Despriet, D. D. G., Klaver, C. C. W., & Simonsz, H. J. (2016). Patient-reported utilities in bilateral visual impairment from amblyopia and age-related macular degeneration. *BMC Ophthalmology, 16*, 56. doi:10.1186/s12886-016-0234-0

van de Graaf, E. S., van Kempen-du Saar, H., Looman, C. W. N., & Simonsz, H. J. (2010). Utility analysis of disability caused by amblyopia and/or strabismus in a population-based, historic cohort. *Graefe's Archive for Clinical and Experimental Ophthalmology, 248*(12), 1803-1807. doi:10.1007/s00417-010-1457-z

Walkinshaw, E. (2011). Mandatory vaccinations: The Canadian picture. *Canadian Medical Association Journal, 183*(16), E1165. doi:10.1503/cmaj.109-3992

Wall, T. C., Marsh-Tootle W Fau - Evans, H. H., Evans Hh Fau - Fargason, C. A., Fargason Ca Fau - Ashworth, C. S., Ashworth Cs Fau - Hardin, J. M., & Hardin, J. M. Compliance with vision-screening guidelines among a national sample of pediatricians. (1530-1567 (Print)).

Wallace, D. K., Repka, M. X., Lee, K. A., Melia, M., Christiansen, S. P., Morse, C. L., & Sprunger, D. T. (2017). *Amblyopia Preferred Practice Pattern*. Retrieved from San Francisco, CA: http://www.aaojournal.org/content/preferred-practice-pattern

Wallace, M. P., Stewart, C. E., Moseley, M. J., Stephens, D. A., & Fielder, A. R. (2013). Compliance with occlusion therapy for childhood amblyopia. *Invest Ophthalmol Vis Sci, 54*(9), 6158-6166. doi:10.1167/iovs.13-11861

© 2023 Asare AO et al. *JAMA Network Open*.
