# Supplementary material for: Cost-effectiveness of Universal School- and Community-Based Vision Testing Strategies to Detect Amblyopia in Children in Ontario, Canada
Source: JAMA Netw Open. 2023 Jan 4;6(1):e2249384. doi: 10.1001/jamanetworkopen.2022.49384 (PMC9857467; doi:10.1001/jamanetworkopen.2022.49384)
Supplement: Supplement 2. — Data Sharing Statement [file jamanetwopen-e2249384-s002.pdf]

Data Sharing Statement

Asare. Cost-effectiveness of Universal School- and Community-Based Vision Testing Strategies to Detect Amblyopia in Children in Ontario, Canada. *JAMA Netw Open*. Published January 04, 2023. doi:10.1001/jamanetworkopen.2022.49384

Data
Data available: No
